# Ultra-high field brain MRI for functional neurological disorder: opportunities and challenges^☆^

**DOI:** 10.1016/j.nicl.2026.103972

**Published:** 2026-02-19

**Authors:** Sverre Myren, Johannes Jungilligens, Ibai Diez, Erlend Bøen, Torbjørn Elvsåshagen, Birte Forstmann, Maryam Ziaei, Thanh P. Doan

**Affiliations:** a Department of Neurology, Ålesund Hospital, Ålesund, Norway; b Clinical Brain Systems (CBS) Group, Department of Neuromedicine and Movement Science, Norwegian University of Science and Technology (NTNU), Trondheim, Norway; c Department of Health Sciences Ålesund, Norwegian University of Science and Technology (NTNU), Ålesund, Norway; d Department of Neurology, University Hospital Knappschaftskrankenhaus Bochum, Ruhr University Bochum, Bochum, Germany; e Center for Inflammation Imaging, Mass General Brigham, Harvard Medical School, Boston, MA, USA; f Computational Neuroimaging Lab, Biobizkaia Health Research Institute, Barakaldo, Spain; g Ikerbasque - Basque Foundation for Science, Bilbao, Spain; h Section of Psychosomatic Medicine, Oslo University Hospital, Oslo, Norway; i Department of Behavioural Medicine, Institute of Basic Clinical Sciences, University of Oslo, Oslo, Norway; j Department of Neurology, Oslo University Hospital, Oslo, Norway; k Centre for Precision Psychiatry, Division of Mental Health and Addiction, Oslo University Hospital, Oslo, Norway; l Department of Psychology, the University of Amsterdam, Amsterdam, the Netherlands; m Kavli Institute for Systems Neuroscience, Norwegian University of Science and Technology (NTNU), Trondheim, Norway; n Department of Neurology and Clinical Neurophysiology, St. Olav’s University Hospital, Trondheim, Norway

**Keywords:** Functional neurological disorder, FND, Ultra-high field MRI, 7T MRI, Biophysical multimodal MRI, Quantitative MRI

## Abstract

Functional neurological disorder (FND) is a common condition with debilitating neurological symptoms emerging from brain network alterations; yet, neuroimaging findings are often of unclear neurobiological significance. Contemporary mechanistic accounts indicate disturbances in hierarchical brain processing involving arousal, interoception, salience attribution, affective-motor integration, and self-agency. The findings of abnormal brain function with only partially consistent neuroimaging abnormalities imply that relevant pathology may occur below the resolution or contrast capabilities of conventional clinical MRI. Ultra-high field MRI (UHF, 7T and above) provides substantially improved spatial resolution, contrast sensitivity, and physiological specificity needed to interrogate microcircuit-, mesoscale-, and neurochemical-level mechanisms. We review how UHF-enhanced structural, diffusion, functional, and spectroscopic techniques can address long-standing mechanistic questions in FND. Selected targets that operate at different spatial scales are used to illustrate how UHF MRI can resolve features that are either inaccessible or not fully reliable at standard magnetic field strengths (1.5–3T). To synthesize these advances, we propose a biophysical multimodal MRI framework that integrates quantitative MRI, diffusion MRI, functional MRI, and MR spectroscopy to derive biologically interpretable markers spanning microstructure, white matter architecture, circuit dynamics, and neurometabolic domains. This approach aligns with contemporary computational and neurobiological models of FND and offers a scalable roadmap for future research. Together, UHF MRI and biophysically grounded multimodal imaging can enable a new generation of mechanistic studies in FND, with potential implications for diagnosis, subtype stratification, and biomarker development.

## Highlights

• FND arises from brain network dysfunctions, yet imaging findings are inconsistent.
• UHF MRI offers higher sensitivity to reveal alterations underlying FND.
• We outline the opportunities and challenges of applying UHF MRI to FND research.
• We propose a biophysical multimodal MRI framework capturing FND symptom construction.
• Most of the underlying principles are also applicable at standard field strengths.

## Graphical abstract

**Figure:**
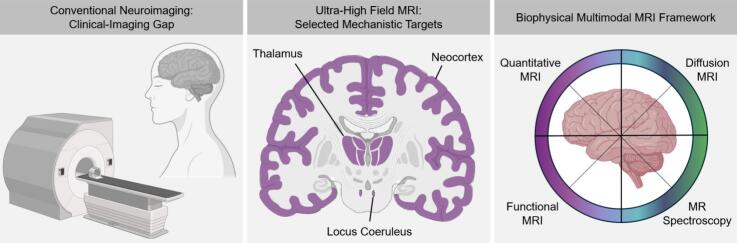

## 1 Introduction

### 1.1 A clinical–imaging gap

Functional neurological disorder (FND) is among the most disabling yet least understood neurological syndromes. According to DSM-5/DSM-5-TR (Diagnostic and Statistical Manual of Mental Disorders, Fifth Edition, Text Revision (DSM-5-TR) 2022), FND mimics symptoms of other neurological disorders but lacks their typical functional and structural brain correlates. Common presentations include functional movement disorders, functional/dissociative seizures, functional sensory deficits, functional communication and swallowing disorders, functional cognitive disorder, and functional dizziness (persistent postural-perceptual dizziness) (Aybek and Perez, 2022).

Structural brain abnormalities are not uncommon in FND (Perez et al., 2021). However, most findings are nonspecific and cannot be linked to the symptoms reported by the patient, or do not account for comorbidities that may also be associated with structural abnormalities. In contrast, functional neuroimaging more consistently demonstrates altered activity and connectivity within cortico-subcortical circuits governing emotion, attention, salience, and motor control (Hallett et al., 2022, Perez et al., 2021). These findings of abnormal function with only partially consistent structural abnormalities imply that relevant pathology may occur below the resolution or contrast capabilities of conventional clinical MRI (Bègue et al., 2019). Biophysically informed multimodal MRI approaches – such as quantitative MRI (qMRI), diffusion MRI (dMRI), functional MRI (fMRI), and MR spectroscopy (MRS) – provide biologically interpretable metrics that probe microstructural, physiological, and molecular features not visible on standard clinical imaging. Ultra-high-field (UHF, 7T and above) systems may further enhance the sensitivity to detect pathological alterations that are hitherto not detected. Yet, UHF MRI has not been applied to the study of FND. In this narrative review, we aim to highlight the opportunities and challenges of UHF MRI and the biophysical multimodal MRI framework in FND research. These tools should be used in a hypothesis-guided approach to disentangle the pathomechanisms underlying FND. We first provide an overview of mechanistic models of FND and their implications for selected regions of interest within the underlying functional anatomy.

### 1.2 Mechanistic models of FND

Converging neuroimaging, experimental, and theoretical work suggests that FND arises from alterations in attention (e.g., misdirected attention towards bodily sensations), emotion (e.g., difficulties in understanding and regulating emotion states), sensorimotor function (e.g., loss of control over movements), and bodily awareness (e.g., reduced perception and integration of interoceptive signals) (Drane et al., 2020, Perez et al., 2021). The contemporary pathomechanistic understanding assumes alterations in predictive inference that affect these interconnected functions rather than assuming a single lesion or network defect (Paredes-Echeverri et al., 2022, Hallett et al., 2022, Jungilligens and Perez, 2025). In line with these potential brain-wide network effects, several partially overlapping systems-level hypotheses have been formulated as contributing to FND pathomechanisms:

1) altered allostasis and arousal dysregulation,
2) abnormal filtering of interoceptive and sensorimotor signals,
3) salience misallocation and altered attention to bodily signals,
4) altered emotion construction with strong bodily symptoms/movement components,
5) impaired sense of agency, and
6) – as an overarching mechanism relevant throughout all previous points – predictive coding imbalance with top-down priors outweighing bottom-up signals, as well as altered prediction error integration, producing false inferences.

These pathomechanistic hypotheses allow us to infer potentially relevant brain networks and regions of interest for investigation through advanced neuroimaging studies (Table 1), for which we outline selected examples here:

**Table 1:** Convergence of functional neuroanatomy and pathophysiological mechanisms: examples of supportive neuroimaging evidence and UHF MRI advantages.

**Brain region(s)**	**Pathomechanism**						**Examples of supportive neuroimaging evidence in FND**	**UHF MRI advantages**
	1	2	3	4	5	6		
SMA		(•)			•	•	Increased SMA activation (Aybek et al., 2015, Aybek et al., 2014) and increased SMA-amygdala connectivity during emotional task fMRI (Voon, Brezing, et al., 2010).	Dissection of feedback (top-down) and feed-forward (bottom-up) hierarchical signaling using layer-specific fMRI (Haarsma and Kok 2025).
ACC	(•)		•			•	Increased cingulate gyrus activation during emotional task fMRI (Aybek et al., 2015). Altered cingulo-insula and amygdala activation during motor behavior and at rest (Ospina et al., 2019, Voon et al., 2011).	
rTPJ			(•)		•	•	Abnormal rTPJ activity, most commonly reported to be decreased (Nahab et al., 2017, Bühler et al., 2024, Weber et al., 2025, Voon et al., 2010b). Decreased rTPJ connectivity with sensorimotor and limbic regions during task fMRI (Voon, Gallea, et al., 2010).	
Insula	(•)	(•)	•			•	Decreased activation in right anterior insula, correlating with interoceptive accuracy (Sojka et al., 2025). Impaired functional insular-rTPJ connectivity during rs-fMRI (Maurer et al., 2016). Stronger functional connectivity between insular subregions and sensorimotor network, lingual gyrus, superior parietal gyrus and putamen (Li et al., 2015). Enhanced stepwise connectivity between right laterobasal amygdala and left anterior insula (Diez et al., 2019).	
Amygdala			•	•		•	Amygdala hyperactivity during emotional and sensorimotor tasks (Aybek et al., 2015, Voon et al., 2010a, Hassa et al., 2017). Increased amygdala-motor connectivity (Aybek et al., 2014). Structural MRI most typically shows increased amygdala volume (Perez et al., 2017, Maurer et al., 2018), yet smaller volume was also reported (Weber et al., 2023).	Enhanced visualization of the amygdala and its internal architecture at 7T (Derix et al., 2014). Ultra-high-resolution reference atlases have been implemented in automated tools such as FreeSurfer (Saygin et al., 2017).
Extended basal ganglia system		•				•	Right caudate-amygdala connectivity best discriminated motor FND patients from controls in a resting-state fMRI study (Wegrzyk et al., 2018). Structural changes with basal ganglia volume loss (Atmaca et al., 2006).	Detailed visualization of all major nuclei, including the caudate, putamen, globus pallidus, subthalamic nucleus, substantia nigra, and nucleus accumbens. Submillimeter voxel sizes allow clear delineation of internal architecture such as the GPi/GPe boundary and striatal compartments. A widely used probabilistic atlas derived from 7T MRI provides high-precision anatomical reference maps (Keuken and Forstmann 2015). Voxel sizes as small as 0.5 mm^3^ achievable, allowing fine delineation of subthalamic nucleus (STN) borders. Susceptibility-based contrasts (e.g., T2*, SWI, QSM) highlight iron-rich STN tissue (de Hollander et al., 2017). Increased SNR and enhanced susceptibility contrast improve substantia nigra (SN) delineation from adjacent midbrain tissue (Brammerloh et al., 2022).
Hippocampal formation	(•)					•	Decreased left hippocampal activity during task fMRI paradigm examining neural correlates of recall of life events (Aybek et al., 2014). Reduced hippocampal and amygdala volumes was reported (Weber et al., 2023).	Direct visualization of the hippocampal stratum radiatum-lacunosum moleculare (SRLM) as a hypointense band on T2W (Kerchner et al., 2012). SRLM serves as a key landmark to delineate hippocampal subfields manually (DG, CA3, CA2, CA1, Sub) in tilted coronal section with in-plane resolution superior to 0.5 mm x 0.5 mm, with high inter- and intrarater reliability (Berron et al., 2017).
Thalamus	•	•	•	•	•	•	Reduced left thalamic volume in motor FND compared to controls (Nicholson et al., 2014). SPECT studies: Reduced regional cerebral blood flow in the thalamus and basal ganglia contralateral to the functional sensorimotor deficit (Vuilleumier et al., 2001).	7T allows manual delineation of up to 15 nuclei based on directly visible medullary lamellae rather than atlas warping (Tourdias et al., 2014). 7T substantially enhances nuclei separability (Segobin et al., 2024) and supports the development of automated segmentation tools such as *THOMAS*.
PAG	•		•			•	Hyperactivation during negative emotion task fMRI in motor FND (Aybek et al., 2015). Enhanced PAG-amygdala connectivity (Diez et al., 2019). Increased PAG volume in subgroups (Perez et al., 2017).	3T studies include mapping PAG structural connectivity (Cacciola et al., 2019) by warping a probabilistic 7T atlas into individual 3T spaces (Keuken and Forstmann 2015), or generating subject-specific PAG masks for connectivity-based segmentation (Ezra et al., 2015). At 7T, an automated parcellation tool has recently become available (Bazin et al., 2025), illustrating the enhanced specificity achievable at UHF.
LC	•		(•)			•	Higher state persistence in LC co-activation patterns, suggesting altered arousal-state dynamics (Weber et al., 2024). Volume loss in brainstem-forebrain monoaminergic nuclei including LC, accompanied by prolonged states of limbic, motor, and interoceptive region hyperconnectivity (Mueller et al., 2024).	A 3D magnetization-transfer turbo-FLASH (MT-TFL) sequence enable sub-millimeter isotropic imaging of the LC at both 3T and 7T (Priovoulos et al., 2018). At 7T, MT-TFL provides two- to four-fold finer resolution (0.5 mm vs 1–2 mm), improved SNR, and shorter acquisitions, offering the most precise LC delineation currently achievable.
DRN	•	(•)				•	Simultaneous PET and 3T fMRI in healthy volunteers revealed that higher BOLD response in the amygdala correlated with reduced DRN serotonin availability while viewing emotional faces (Janet et al., 2023). Dynamic-fMRI work at 3T demonstrated that patients with functional/dissociative seizures show volume loss in brainstem-forebrain monoaminergic and cholinergic nuclei (including LC, PAG, and raphe nuclei) accompanied by prolonged states of limbic, motor, and interoceptive region hyperconnectivity (Mueller et al., 2024).	Emerging ultra-high field imaging methods now offer more precise access to raphe nuclei *in vivo*. A probabilistic 7T template of mesopontine tegmental nuclei (Bianciardi et al., 2018) has been expanded into comprehensive probabilistic atlases (Singh, García-Gomar, and Bianciardi 2021). More recently, DRN has also been included in probabilistic atlases (Bazin et al., 2025).
Cerebellum		•		(•)	•	•	Impaired rTPJ-cerebellar functional connectivity reported in a rs-fMRI study (Maurer et al., 2016). Increased activation of cerebellar-limbic network during task fMRI examining effect of negative emotions on isometric precision-grip force output (Blakemore et al., 2016). Cerebellar volume loss is most commonly reported in functional/dissociative seizures (McSweeney et al., 2017, Labate et al., 2012), while increased volume has been reported in motor FND (Maurer et al., 2018).	Emerging ultra-high field methods demonstrated an enhanced detail of cerebellar cortex using 7T MP2RAGE compared to FLASH sequence. The cortical surface area was 1.8 times larger, and the cortical thickness was five times thinner than previous *in vivo* estimates, which is closer to *ex vivo* reference data (Priovoulos et al., 2023). The usability was demonstrated in patients with early-stage multiple sclerosis (Fartaria et al., 2017).

Direct and indirect evidence suggests that distributed cortical and subcortical regions are involved in core FND pathomechanisms. Most of the selected regions of interest in this table are well established in the literature, while others remain less studied, likely due in part to methodological challenges associated with investigating them. The list is not exhaustive. We have outlined six core pathomechanisms: (1) Altered allostasis and arousal dysregulation, (2) abnormal filtering of interoception and sensorimotor signals, (3) salience misallocation and altered attention to bodily signals, (4) altered emotion construction with strong bodily symptoms/movement components, (5) impaired sense of agency, and (6) predictive coding imbalance, which serves as an overarching framework for all the above mechanisms. The correspondence of brain regions and these pathomechanisms is weighted as follows: •: indicates strong evidence. (•): indicates weaker evidence. *Abbreviations: ACC, anterior cingulate cortex; BOLD, blood-oxygenation-level-dependent; CA (1*–*3), cornu ammonis field (1*–*3); DG, dentate gyrus; DRN, dorsal raphe nucleus; FLASH, fast low-angle shot; GPi/GPe, internal/external globus pallidus*; *LC, locus coeruleus; ls-fMRI, layer-specific fMRI; MP2RAGE, magnetization prepared 2 rapid acqusition gradient echoes; MT-TFL, magnetization-transfer turbo-FLASH; PAG, periaqueductal gray; PET, positron emission tomography; QSM, quantitative susceptibility mapping; rs-fMRI, resting-state fMRI; rTPJ, right temporo-parietal junction; SMA, supplementary motor area; SN, substantia nigra; SNR, signal-to-noise-ratio; SPECT, single-photon emission computed tomography; SRLM, stratum radiatum-lacunosum moleculare; STN, subthalamic nucleus; Sub, subiculum; SWI, susceptibility-weighted imaging; THOMAS, Thalamus Optimized Multi Atlas Segmentation*.

#### 1.2.1 Altered allostasis and arousal dysregulation

Predictive body regulation through allostasis is a brain-wide process (Sterling, 2012, Theriault et al., 2025). When considered within the framework of arousal dysregulation (Paredes-Echeverri et al., 2022), this process highlights how maladaptive locus coeruleus (LC)-noradrenergic and raphe-serotonergic output can destabilize network states and perturb key regions involved in memory (e.g., hippocampus) and interoception (e.g., anterior cingulate cortex [ACC], and insula). Supporting this view, recent dynamic-fMRI work at 3T demonstrated that patients with functional/dissociative seizures show volume loss in brainstem-forebrain monoaminergic and cholinergic nuclei (including LC, periaqueductal grey [PAG], and raphe nuclei) accompanied by prolonged states of limbic, motor, and interoceptive region hyperconnectivity (Mueller et al., 2024). Additionally, higher state persistence of locus coeruleus-related co-activation patterns was shown in patients with functional/dissociative seizures in regions closely overlapping with the default mode network (Weber et al., 2024). The LC and its potential role in FND are discussed in more detail in a subsequent section (section 2.1.1).

#### 1.2.2 Abnormal filtering of interoceptive and sensorimotor signals

In many patients with FND, symptom occurrence is related to autonomic arousal and potentially to misrepresentation of interoceptive arousal signals (Paredes-Echeverri et al., 2022, Millman et al., 2023, Sojka et al., 2025). For example, cortical processing of interoceptive signals was reduced during functional/dissociative seizures, consistent with an abnormal filtering of interoceptive and sensorimotor signals (Elkommos et al., 2023, Flasbeck et al., 2024). Fittingly, in motor FND, one study demonstrated abnormal interoceptive processing in a network involving the precuneus, the posterior cingulate cortex and caudate nucleus bilaterally and the right anterior insula (Spagnolo et al., 2025), and another study revealed individual differences in interoceptive accuracy and interoceptive trait prediction error correlated with fiber bundle integrity originating from the insula and thalamus among other regions (Sojka et al., 2021). A recent study using graph theory based resting-state fMRI (rs-fMRI) to compare FND patients to psychiatric controls additionally found increased crosstalk between the cortical portions of the somatomotor network with cortical portions of the salience, default mode, and frontoparietal networks, implying a cortico-cortical disturbance of sensorimotor signals (Westlin, Guthrie, Bleier, Finkelstein, Maggio, Ranford, MacLean, Godena, Millstein, Paredes-Echeverri, et al., 2025). Overall, sensorimotor signals are assumed to be distorted in FND, which can be conceptualized as abnormal basal ganglia-thalamic gating, i.e., abnormal “filtering” of limbic/motor signals via basal ganglia laminae.

#### 1.2.3 Salience misallocation and altered attention to bodily signals

Salience misallocation and altered attention to bodily signals represent additional critical aspects of FND pathomechanistic models. Patients often show heightened attention to potentially threatening bodily sensations, such that normal physiological fluctuations may be misinterpreted as signs of pathology. This pattern reflects altered interoceptive modelling; specifically, salience signaling within the salience network (i.e., anterior insula and ACC) appears to be misallocated, leading to a distorted weighing of interoceptive relative to exteroceptive input. Evidence from both functional and structural neuroimaging supports this view. Functional imaging consistently reports alterations and hyperconnectivity within salience network regions (van der Kruijs et al., 2012, van der Kruijs et al., 2014, Weber et al., 2022, Aybek et al., 2015, Diez et al., 2019, Li et al., 2015), while structural imaging demonstrated associations between salience network gray matter volumes and symptom severity (Jungilligens et al., 2022a, Perez et al., 2018).

#### 1.2.4 Altered emotion construction with strong bodily symptoms/movement components

Altered emotion construction with strong bodily symptoms or movement components has been posited as a key explanation for the occurrence of functional motor symptoms (Jungilligens, Paredes-Echeverri, et al., 2022). Early functional neuroimaging studies in FND provide supporting evidence for an altered link between emotion and movement: excessive amygdala activation in response to emotional stimuli has been shown to drive increased activity in motor regions (Aybek et al., 2015, Voon et al., 2010a). FND patients also exhibited a correlation between symptom severity and enhanced functional connectivity of the left anterior insula, the right anterior insula, and the temporoparietal junction (TPJ) (Diez et al., 2019), indicating a symptom-related link between insula-mediated affective processes and TPJ-mediated sense of agency processes. These neuroimaging studies support the notion of systematic alterations in emotion construction in FND, as also evidenced by the repeated findings of higher alexithymia levels (Ostuzzi et al., 2025) or the notion of “panic without panic” in patients with functional/dissociative seizures (Goldstein and Mellers, 2006).

#### 1.2.5 Impaired sense of agency

Patients with FND report impaired sense of agency (i.e., loss of subjective willful control of their actions) with respect to their symptoms. This is related to a breakdown of the predictive-matching process between intended motor commands (supplementary motor area [SMA]/pre-SMA) and sensory feedback processed in the right temporoparietal junction (rTPJ), causing actions to feel involuntary or externally controlled despite being self-produced (Maurer et al., 2016, Nahab et al., 2017, Hallett et al., 2022, Kranick et al., 2013, Baek et al., 2017). This pattern has also been found in children with FND: across development, self-agency circuitry shows convergent disruptions with reduced resting-state connectivity between anterior medial prefrontal cortex and the rTPJ ((Walpola et al., 2025), this issue).

#### 1.2.6 Predictive coding imbalance

Across all of the above points, predictive processing frameworks offer fundamental mechanistic insights into the pathophysiology of FND, and imbalances in predictive coding have been put at the core of FND symptoms (Hallett et al., 2022, Edwards et al., 2012; Paredes-Echeverri et al., 2022, Jungilligens and Perez, 2025) – supported by several studies that provide experimental evidence of aberrant predictive coding in motor learning, sensory judgement, and attention (Pareés et al., 2012a, Lin et al., 2020, Huys et al., 2021, Sadnicka et al., 2020, Pareés et al., 2012b). Fundamentally, imbalances are assumed with top-down priors outweighing bottom-up signals as well as altered prediction error integration, producing false inferences. At the network level, a dynamic “overshooting” brain state was identified in functional/dissociative seizures, characterized by hyperconnectivity between emotional-control hubs (subgenual, anterior, and mid cingulate, dorsolateral frontal, and insular cortices) and body-ownership/agency regions (SMA, postcentral gyrus, superior parietal lobule) (Mueller et al., 2024). This state emerged alongside volume loss in brainstem and forebrain monoaminergic/cholinergic nuclei (LC, PAG, and raphe nuclei), suggesting that dysregulated neuromodulatory systems fail to constrain ascending prediction errors. This view was extended by a study in patients with functional/dissociative seizures, demonstrating that LC-related co-activation patterns in regions closely overlapping with the default mode network showed higher state persistence (Weber et al., 2024). Together, these findings imply that impaired neuromodulatory gating permits excessive error signaling that influences the default mode network dynamics, modulating the weighting of self-related priors and jointly disrupting the hierarchical inference processes that underpin sense of self and self-agency. While these are brain-wide processes, the details of predictive processing streams are reflected in the cortical lamination profiles, with thalamic relays, LC, and salience network precision systems additionally weighting predictions and prediction errors. A recent study provided the first evidence for altered hierarchical cortical organization in FND (Westlin, Guthrie, Bleier, Finkelstein, Maggio, Ranford, MacLean, Godena, Millstein, Freeburn, et al., 2025).

## 2 Regional examples highlighting UHF MRI advantages

**Fig. 1:**
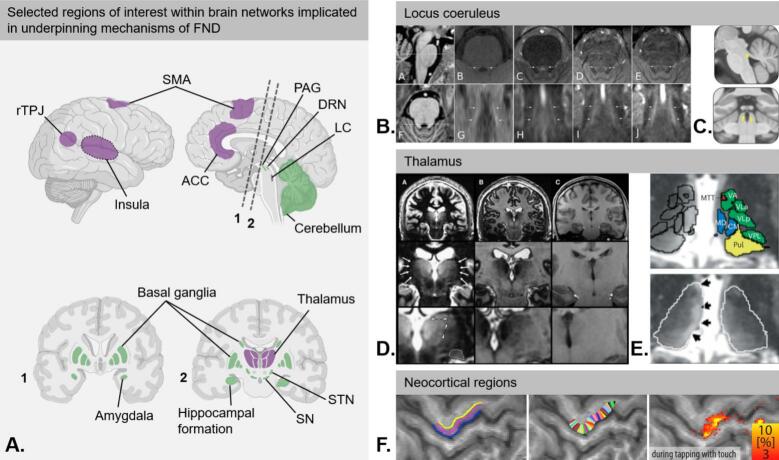
Selected regions of interest: functional neuroanatomy and prospective UHF MRI utility. A. Selected regions of interest (ROIs) implicated in underpinning mechanisms of FND (for details, see Table 1). Three selected ROIs, which operate at different spatial scales, are highlighted in purple: a tiny subcortical nucleus (the locus coeruleus, LC), a larger subcortical structure (the thalamus), and neocortical regions (rTPJ, SMA, ACC, and insula). All remaining selected regions from Table 1 are shown in green color. B. The locus coeruleus is reliably visualized at 7T MRI using a 3D magnetization-transfer turbo-FLASH (MT-TFL) sequence (Priovoulos et al., 2018). Arrows indicate the LC as a hyperintense structure, throughout scans in the same participant. Upper panels show axial sections through the pons, at the level shown in (A); lower panels show coronal slices at the level given in (F). From left to right, axial sections (B-E) and coronal sections (G-J), respectively, are displayed for 3T TSE, 3T MT-TFL, 7T single acquisition, and 7T MT-TFL 2-average. The MT-TFL showed contrast in the LC region both at 3T and 7T, yet the contrast intensity is more pronounced at 7T MRI. C. 7T MT-TFL sequence used to delineate LC during a facial emotion recognition task across the lifespan (in yellow, normalized into MNI template space (Dave et al., 2025)). D. 7T MRI substantially enhances thalamic nuclei separability. Optimized white matter (WM)-nulled magnetization-prepared rapid acquisition gradient-echo (MPRAGE) with measured T1 values enables continuous visualization of the internal medullary laminae, allowing manual delineation of up to 15 nuclei. Panels show acquisition utilizing the white matter (WM) null regime (A), gray matter (GM) null regime (B), and a standard MPRAGE protocol (C). Arrows indicate the higher signal and improved delineation of the external boundaries of the thalamus (A), and dotted lines at higher magnification delineation of the lateral geniculate nucleus from the surrounding white matter (Tourdias et al., 2014). E. Automated segmentation tool THOMAS (Thalamus Optimized Multi Atlas Segmentation) has already demonstrated clinical utility by detecting nucleus-specific atrophy in multiple sclerosis. Masks for 12 individual thalamic nuclei were generated (upper panel), in which lesions are indicated with arrows along the ependymal surface (lower panel) (Planche et al., 2020, Su et al., 2019). F. Layer-specific fMRI (ls-fMRI) enables interrogation of hierarchical information flow in cortical circuits. In a finger-tap task ls-fMRI study, primary motor cortex (M1) was separated into superficial, middle, and deep laminae (left panel) and columns (middle panel), demonstrating layer-specific BOLD-signal fluctuations (right panel) (Huber et al., 2017). Figure A adapted from BioRender. Remaining figures adapted and reproduced with permission. *Abbreviations: ACC, anterior cingulate cortex; BOLD, blood-oxygenation-level-dependent imaging; DRN, dorsal raphe nucleus; GM, gray matter; LC, locus coeruleus; ls-fMRI, layer-specific fMRI; MNI, Montreal Neurological Institute; MPRAGE, magnetization-prepared rapid acquisition gradient-echo; MT-TFL, magnetization-transfer turbo-FLASH; PAG, periaqueductal gray; ROI, region of interest; rTPJ, right temporo-parietal junction; SMA, supplementary motor area; SN, substantia nigra; STN, subthalamic nucleus; THOMAS, Thalamus Optimized Multi Atlas Segmentation; TSE, turbo spin echo; WM, white matter.* (For interpretation of the references to colour in this figure legend, the reader is referred to the web version of this article.)

The pathophysiology of FND is increasingly understood as emerging from network dysfunctions rather than from focal pathological alterations. Nevertheless, several anatomical regions appear to play disproportionate roles in key mechanistic processes. To illustrate how UHF MRI can advance mechanistic understanding in FND, we highlight selected regions of interest (ROIs) that operate at different spatial scales: tiny subcortical nuclei (the LC, the PAG, and other brainstem nuclei), larger subcortical structures (the thalamus, and the amygdala), and cortical regions (the hippocampal formation, and the neocortex). We also summarize additional relevant ROIs involved in mechanistic models (Fig. 1 and Table 1). Each region poses distinct imaging challenges for which UHF MRI confers substantial advantages. By offering superior spatial resolution, tissue contrast, and neurochemical sensitivity, UHF MRI enables more precise characterization of small nuclei, mesoscale architecture, and laminar features that are essential for linking functional anatomical substrates to core pathophysiological mechanisms.

### 2.1 Small subcortical nuclei

#### 2.1.1 The locus coeruleus

As a central hub in the brain’s ascending arousal system, the locus coeruleus is the major source of cortical and subcortical noradrenaline. Through its widespread projections to the amygdala, hippocampus, insula, thalamus, cerebellum, and prefrontal cortex, the LC regulates threat appraisal, interoception, attention, and motor readiness. By dynamically tuning neural gain, it biases perception and cognition toward salient or uncertain stimuli while supporting the consolidation of emotionally relevant information (Poe et al., 2020). Emerging neuroimaging evidence supports LC involvement in FND. In a recent resting-state 3T fMRI study, Weber et al., demonstrated that in patients with functional/dissociative seizures, phasic LC activity-dependent brain activity showed higher state-persistence for default mode network regions, suggesting altered arousal-state dynamics and reduced flexibility of LC-driven network transitions (Weber et al., 2024).

LC imaging has evolved from indirect post-mortem anatomical studies to a sophisticated, non-invasive tool capable of assessing LC integrity *in vivo* (Betts et al., 2019). Early developments showed that MRI protocols sensitive to neuromelanin, a paramagnetic pigment accumulating in LC neurons, could visualize the LC as a hyperintense signal on T1-weighted and magnetization-transfer (MT) images. Early LC MRI studies at 3T relied on 2D T1-weighted turbo spin echo (TSE) (Sasaki et al., 2006). However, their relatively thick slices, limited through-plane resolution, long scan times, and high specific absorption rate (SAR) constrained spatial fidelity and precluded 7T use. Advances in high-resolution MRI (3T and 7T) and optimized voxel design now allow reliable delineation of the LC’s small structure. Priovoulos et al., introduced a 3D magnetization-transfer turbo-FLASH (MT-TFL) sequence that markedly reduced SAR and enabled sub-millimeter isotropic imaging at both 3T and 7T (Fig. 1B-C) (Priovoulos et al., 2018). At 7T, MT-TFL provides two- to four-fold finer resolution (0.5 mm versus 1–2 mm), improved signal-to-noise (SNR), and shorter acquisitions, offering the most precise LC delineation currently achievable. Multimodal validation combining histology, post-mortem MRI, and positron emission tomography (PET) has confirmed that neuromelanin-MRI contrast reflects neuronal density and noradrenergic integrity. 7T MT-TFL has already demonstrated sensitivity to arousal-related LC activity in healthy individuals across the lifespan (Dave et al., 2025).

#### 2.1.2 The periaqueductal gray and other brainstem nuclei

The periaqueductal gray, which encircles the midbrain aqueduct, plays central roles in coordinating integrated behavioral responses to internal (e.g., pain) or external (e.g., threat-related) stressors. This functional diversity is reflected in its organization into four longitudinal columns with distinct neuromodulatory influences (Zhang et al., 2024, Behbehani, 1995). In motor FND, task-based fMRI studies have demonstrated hyperactivation of the PAG during negative emotion processing (Aybek et al., 2015), and resting-state fMRI has shown enhanced PAG-amygdala connectivity (Diez et al., 2019). Structural alterations have also been reported, with increased PAG volume demonstrated in specific FND subgroups (Perez et al., 2017). At 3T, PAG delineation has included structural connectivity mapping (Cacciola et al., 2019) using probabilistic 7T atlases warped into individual 3T spaces (Keuken and Forstmann, 2015), as well as subject-specific PAG masks for connectivity-based segmentation (Ezra et al., 2015). More recently, 7T MRI approaches include manual delineation of PAG subregions during respiratory or threat paradigms (Weis et al., 2022, Faull et al., 2015) as well as automated parcellation tools (Bazin et al., 2025), illustrating the enhanced specificity achievable at UHF.

Direct supportive neuroimaging evidence in FND remains limited for the dorsal raphe nucleus (DRN) and many other brainstem nuclei. The DRN is a heterogeneous midbrain structure that constitutes a major source for serotonin, a neuromodulator centrally involved in the regulation of emotional states (Dorocic et al., 2014, Jacobs and Azmitia, 1992). Abnormalities in serotonin synthesis, together with strong serotonergic control of amygdala circuits, suggest a potential role in the emotion dysregulation observed in FND (Hallett, 2024, Sengupta et al., 2017). In healthy volunteers, simultaneous PET and 3T fMRI revealed that greater amygdala blood-oxygen-level-dependent (BOLD) responses during emotional face processing correlated with reduced DRN serotonin availability (Janet et al., 2023). In patients with functional/dissociative seizures, dynamic fMRI at 3T has shown volume loss in brainstem-forebrain monoaminergic and cholinergic nuclei (including LC, PAG, and raphe nuclei) that was accompanied by prolonged states of limbic, motor, and interoceptive region hyperconnectivity (Mueller et al., 2024). Emerging ultra-high field imaging approaches now offer improved *in vivo* access to raphe nuclei. A probabilistic 7T template of mesopontine tegmental nuclei (Bianciardi et al., 2018) has been subsequently expanded into comprehensive probabilistic atlases (Singh, García-Gomar, and Bianciardi, 2021). More recently, the DRN has also been included in probabilistic atlases (Bazin et al., 2025).

### 2.2 Larger subcortical structures

#### 2.2.1 The thalamus

The thalamus is a bilateral subcortical structure within the diencephalon, comprising numerous nuclei with distinct connectivity patterns (Segobin et al., 2024). These nuclei predominantly project to specific extra-thalamic areas rather than to each other, indicating that the thalamus should not be treated as a uniform functional unit. The thalamus is no longer regarded as a mere passive ‘relay station’, but is now widely recognized as a critical hub for integrating cortical and subcortical networks, contributing to diverse brain functions including arousal, attention, motor coordination, working memory, and sleep-wake regulation (Segobin et al., 2024, Shine et al., 2023). While evidence supports nuclei-specific specialization, these roles appear to reflect domain-general contributions rather than strictly isolated cognitive or behavioral functions (Shine et al., 2023). Although the thalamus is implicated in all core pathomechanisms of FND – such as imprecise predictive coding (Kanai et al., 2015, Rikhye et al., 2018), attentional processing (Zhou et al., 2016, Snow et al., 2009, Saalmann et al., 2012, Kanai et al., 2015), and altered emotion construction (Paredes-Echeverri et al., 2022, Timbie and Barbas, 2015) – direct neuroimaging evidence in FND remains limited. Structural MRI studies have reported reduced left thalamic volume compared to controls, interpreted either as a primary pathological feature or a secondary consequence of limb disuse (Nicholson et al., 2014). Single-photon emission computerized tomography (SPECT) has shown a decreased regional cerebral blood flow in the thalamus and basal ganglia contralateral to the functional sensorimotor deficit, with this hypoactivation normalizing after clinical recovery (Vuilleumier et al., 2001).

Neuroimaging delineation of thalamic nuclei remains challenging: although approximately 40 nuclei have been defined histologically, only a subset is represented in current neuroimaging atlases, limiting nuclei-specific studies (Segobin et al., 2024, Shine et al., 2023). UHF MRI offers clear advantages for studying the thalamus by providing the spatial resolution and contrast needed to resolve its complex internal architecture. At 7T, submillimeter voxel sizes enable precise delineation of thalamic nuclei that are not reliably distinguishable at standard field strengths. Optimized white matter (WM)-nulled magnetization-prepared rapid acquisition gradient-echo (MPRAGE) with measured T1 values enables continuous visualization of the internal medullary laminae, allowing manual delineation of up to 15 nuclei rather than atlas warping (Tourdias et al., 2014). A related 3T WM-nulled approach offers improved intra-thalamic contrast but only partial lamina visibility, making atlas-guided labeling necessary (Bender et al., 2011). Subsequent work has shown that 7T substantially enhances nuclei separability (Fig. 1D-E) (Segobin et al., 2024) and supports the development of automated segmentation tools such as *THOMAS* (Thalamus Optimized Multi Atlas Segmentation), which has already demonstrated clinical utility by detecting nucleus-specific atrophy in multiple sclerosis (Su et al., 2019, Planche et al., 2020). Future research may establish precise links between specific thalamic nuclei and pathomechanisms underlying FND. For example, midline (e.g., paraventricular) and ventral (e.g., ventromedial) nuclei may play a particularly important role in interoceptive information pathways (Machen et al., 2026, Wen et al., 2021). The ventral thalamic nuclei – including ventromedial, ventrolateral, and ventral anterior nuclei – are also involved in the planning, initiation, and coordination of movement (Sieveritz, García-Muñoz, and Arbuthnott, 2019). In addition, the anterior nuclei and their connections with amygdala and other limbic structures have been implicated in dysfunctional emotion processing (Grodd et al., 2020). Finally, alterations in output from the lateral and posterior thalamic nuclei to the right temporoparietal junction may contribute to disturbances in the sense of agency (Fabio et al., 2022, Krall et al., 2015).

#### 2.2.2 The amygdala

The amygdala is regarded as an important hub for emotion processing through its integration of cortical and subcortical systems (Pessoa, 2017, Pessoa and Adolphs, 2010, LeDoux, 2000). In FND, fMRI studies have demonstrated amygdala hyperactivity during emotional and sensorimotor tasks (Aybek et al., 2015, Voon et al., 2010a, Hassa et al., 2017), alongside increased functional connectivity between the amygdala and motor regions (Aybek et al., 2014). Structural MRI findings have most commonly reported increased amygdala volume (Perez et al., 2017, Maurer et al., 2018), although smaller amygdala volume has also been observed in some cohorts (Weber et al., 2023). 7T MRI enhances visualization of the amygdala and its internal architecture. Compared with 3T, higher field strength allows the amygdalo-hippocampal boundary to be resolved with significantly greater clarity, improving the accuracy of amygdala segmentation and subregional analysis (Derix et al., 2014). Ultra-high-resolution reference atlases, including a 0.1-mm post-mortem amygdala subnuclei atlas constructed using Bayesian inference, have since been implemented in automated segmentation tools such as FreeSurfer (Saygin et al., 2017). Early UHF studies in major depressive disorder demonstrated volumetric alterations in specific amygdala subnuclei linked to symptom severity (Brown et al., 2019).

### 2.3 Cortical regions

#### 2.3.1 The hippocampal formation

The hippocampal formation is a three-layered cortical structure composed of distinct subfields (dentate gyrus, CA3, CA2, CA1, and subiculum). It is crucial for spatial memory and relational memory representations that underlie flexible cognition and social behavior (Eichenbaum, 2017, Rubin et al., 2014). Despite its central cognitive role, the hippocampal formation has received relatively limited attention in FND neuroimaging research. Task-based fMRI examining the neural correlates of autobiographical memory recall has reported decreased left hippocampal activity (Aybek et al., 2014), and structural MRI studies have demonstrated reduced hippocampal and amygdala volumes (Weber et al., 2023). 7T MRI facilitates the visualization of key microanatomical landmarks, such as the stratum radiatum-lacunosum moleculare (SRLM), which appears as a hypointense band on T2-weighted images (Kerchner et al., 2012). By contrast, conventional structural MRI resolutions commonly used for anatomical imaging (e.g., 1.0 mm isotropic) are insufficient for consistent SRLM visualization in oblique coronal sections oriented perpendicular to the hippocampal long axis − an essential prerequisite for reliable hippocampal subfield segmentation (Berron et al., 2017, Wisse et al., 2021).

#### 2.3.2 Neocortical regions

Converging evidence implies the involvement of distributed neocortical circuits in FND (Perez et al., 2021). Neuroimaging studies consistently demonstrate alterations of networks involved in attention, salience, self-agency, interoception, and affective regulation. Within this framework, predictive coding has been proposed as a unifying computational model that integrates these diverse pathomechanisms, helping to account for how aberrant expectations, sensory processing, and agency emerge in FND (Edwards et al., 2012, Jungilligens and Perez, 2025). The laminar architecture of the neocortex provides the structural basis for hierarchical processing of sensation and prediction (Haarsma and Kok, 2025, Jungilligens and Perez, 2025). In predictive coding models, feedback (top-down) signals originate primarily in deep cortical layers and project to deep and superficial layers, while feedforward (bottom-up) signals arise from superficial layers and propagate into the middle layer (layer IV). In FND, it has been suggested that top-down priors may dominate or override incoming (bottom-up) sensory evidence. A recent standard field MRI study provided the first evidence for altered hierarchical cortical organization in FND by demonstrating abnormalities in macroscale cortical gradients (Westlin, Guthrie, Bleier, Finkelstein, Maggio, Ranford, MacLean, Godena, Millstein, Freeburn, et al., 2025), supporting the idea that large-scale hierarchical processing may be disrupted.

UHF MRI is particularly well-suited to interrogate cortical layer dynamics. Its superior SNR ratio and spatial resolution enable sub-millimeter imaging that can resolve fine-grained structures such as cortical layers (Fig. 1F), which are 0.5–1 mm thick (Haarsma and Kok, 2025, Koopmans et al., 2011). Layer-specific fMRI (ls-fMRI) at UHF leverages this resolution to distinguish activity patterns associated with feedforward versus feedback signaling. Studies in other domains have already shown that prediction and prediction-error signals can be layer-specific, for example, in hallucinations or perceptual illusions (Thomas et al., 2024), illustrating the capacity of ls-fMRI to illuminate laminar computations. Translating this methodology to FND could clarify the precise contributions of top-down and bottom-up signaling. For example, layer-specific activation in the ACC or SMA during error detection, volitional movement, or sense of agency tasks could reveal whether abnormal computations originate primarily in deep layers (reflecting altered priors or predictions) or superficial layers (reflecting altered sensory prediction-error signaling) (Haarsma and Kok, 2025).

## 3 Biophysical multimodal MRI framework

**Fig. 2:**
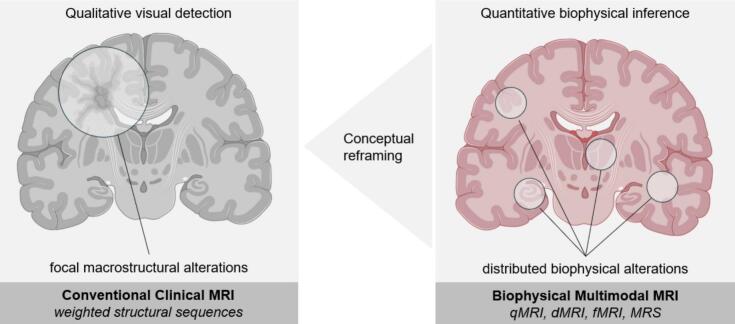
Reframing MRI research in FND: from qualitative radiological assessment to quantitative biophysical inference. Conventional clinical MRI is primarily based on weighted structural sequences optimized for qualitative visual inspection and the detection of focal, radiologically apparent macrostructural abnormalities (left). By contrast, a biophysical multimodal MRI framework integrates quantitative MRI (qMRI), diffusion MRI (dMRI), functional MRI (fMRI), and magnetic resonance spectroscopy (MRS) to enable quantitative inference on distributed microstructural, functional, and metabolic alterations across relevant brain systems (right). Specifically, multiparametric MRI enables quantitative assessment of (1) microstructural tissue integrity using qMRI, (2) white-matter architecture using dMRI, (3) large-scale and laminar functional interactions using fMRI, and (4) neurometabolic state using MRS. Together, these complementary modalities provide convergent, multiscale insights into the distributed biophysical mechanisms that contribute to symptom construction in FND, reflecting a shift in the inferential role of MRI from lesion-focused detection toward systems-level characterization. Figures adapted from BioRender. *Abbreviations: dMRI, diffusion magnetic resonance imaging; fMRI, functional magnetic resonance imaging; MRS, magnetic resonance spectroscopy; qMRI, quantitative magnetic resonance imaging*.

Large retrospective datasets based on standard clinical MRI have been essential for establishing group-level structural patterns in FND (McSweeney et al., 2017, Perez et al., 2021, Bègue et al., 2019, Sasikumar and Strafella, 2021, Hassan et al., 2024). Yet, these macroscopic morphometric measures, while valuable, are limited in their ability to adjudicate among the multiscale mechanisms proposed by contemporary computational and systems-level models. To interrogate hypotheses spanning microstructural properties, white matter signaling, hierarchical functional processing, and neurometabolic signatures, imaging approaches should target finer spatial and physiological scales than those accessible to routine clinical scans. A biophysical multimodal MRI framework provides such an extension. By integrating complementary MRI techniques – including qMRI for microstructural properties, dMRI for white-matter architecture, fMRI for network and laminar dynamics, and MRS for neurometabolic profiling (Fig. 2) – we propose a cardinal framework indicating biologically interpretable markers across multiple complementary scales. While each modality can be implemented at conventional MRI (1.5–3T) field strengths, UHF MRI enhances each domain by improving spatial resolution, contrast, and spectral dispersion. Together, this biophysical multimodal MRI framework approach enables a mechanistic account of how microstructure, connectivity, dynamics, and neurometabolism converge to shape symptom expressions in FND.

### 3.1 Microstructural integrity (qMRI)

Conventional clinical MRI is dominated by weighted sequences (e.g., T1-, T2-, or FLAIR-weighted acquisitions) optimized for the visual assessment of gross abnormalities by radiologists rather than for quantitative biological inference. These contrasts depend strongly on scanner-specific parameters (e.g., gain, coil sensitivity, flip-angle calibration, and B_0_/B_1_ field inhomogeneities), which vary across vendors and acquisition protocols. As a result, two anatomically identical brains can yield different signal intensities purely due to hardware differences, limiting reproducibility in multi-site studies and introducing variability into quantitative analyses. qMRI addresses these limitations by estimating calibrated physical parameters – such as longitudinal relaxation rate (R_1_), effective transverse relaxation rate (R_2_*), proton density (PD), magnetization transfer saturation (MTsat), or magnetic susceptibility (χ) – that are independent of scanner-specific settings and have direct biophysical interpretations (Weiskopf et al., 2021). R_1_ and MTsat are sensitive to myelin and macromolecular composition; R_2_* and χ reflect iron content and microvascular architecture; PD approximates free-water fraction. Together, these measures provide non-invasive *in vivo* histology at the submillimeter scale, offering a window into voxel intensity-specific contributions from myelin loss, dendritic or synaptic density changes, iron redistribution, glial alterations, or extracellular water shifts (Weiskopf et al., 2021). UHF MRI amplifies the power of qMRI: moving from 3T to 7T yields a threefold SNR gain (Pohmann, Speck, and Scheffler, 2016). This substantially improves the precision of R_2_*, quantitative susceptibility mapping (QSM), and related metrics. The resulting surplus SNR enhances susceptibility contrast and enables high-resolution mapping of intracortical myelin gradients, laminar profiles, and small deep-brain nuclei (e.g., thalamic nuclei, LC) implicated in predictive-coding and arousal models of FND. Robust MT-based measures (e.g., MTsat) also benefit from stronger MT effects achievable at 7T. Collectively, UHF qMRI provides a microstructural specificity that is unattainable with conventional 3T morphometry.

Despite this potential, no published FND study has yet applied relaxometry- or susceptibility-based qMRI. Existing structural work relies either on T1-weighted morphometry or on diffusion-based indices (e.g., fractional anisotropy, mean diffusity), which – although quantitative – cannot isolate the biological substrates underlying macrostructural differences. Research in other neurological and psychiatric disorders is emerging to reveal disease-specific evidence for myelin alterations, iron dysregulation, or macromolecular change (Bouhrara et al., 2018, Heij et al., 2024, Vano et al., 2025, Drori et al., 2025). Even in the absence of atrophy or cortical thinning, qMRI is sensitive to subtle microstructural abnormalities that may underlie symptom generation and maintenance. A systematic adoption of qMRI in FND research would entail a shift from macroscopic morphometry to calibrated biophysical interpretable tissue metrics.

### 3.2 Structural connectivity (dMRI)

dMRI provides an *in vivo* assay of white-matter architecture by modelling the microscopic displacement of water constrained by axons and myelin. The widely used diffusion tensor imaging (DTI) model (Basser, Mattiello, and LeBihan, 1994) is fundamentally limited since it estimates only one principal diffusion direction per voxel, despite most white matter voxels (70–90 %) containing crossing fibers (Jeurissen et al., 2013). Given that millimeter-scale voxels contain millions of micrometer-scale axons, DTI cannot resolve this complexity. Motion sensitivity and partial-volume effects further contribute to anatomically implausible tract estimates (Tournier et al., 2008). More advanced methods address these limitations by resolving multiple fiber populations. Constrained spherical deconvolution (CSD) estimates fiber-orientation distributions (FODs), which generate more anatomically valid tractography in regions of complex geometry (Tournier, Calamante, and Connelly, 2007). Beyond geometry, multi-compartment models improve biological interpretability. The most commonly used method – neurite orientation dispersion and density imaging (NODDI) – separates water diffusion in intra- and extra-neurite spaces, providing better proxies for neurite density and dispersion that are more sophisticated than DTI metrics (e.g., fractional anisotropy, mean diffusivity) (Zhang et al., 2012). Although diffusion contrast primarily depends on gradient amplitude rather than field strength, UHF MRI offers indirect benefits through increased SNR. This can be traded for smaller voxels, higher angular resolution, and reduced partial-volume effects, improving tractography near grey–white boundaries and in limbic or temporo-parietal regions where fiber geometry is complex (Gallichan, 2018). These advantages are tempered by challenges at 7T – including B_0_/B_1_ inhomogeneity and susceptibility distortions – which can offset some SNR gains. Importantly, gradient strength remains the principal determinant of diffusion encoding: Connectome-class 3T systems (e.g., 300 mT/m) currently outperform clinical 7T scanners in achievable b-values and angular resolution (Huang et al., 2021).

dMRI has been extensively used in FND research, with studies consistently demonstrating subtle, distributed microstructural alterations in key circuits related to affect regulation, self-agency, and executive control (Hernando et al., 2015, Tomic et al., 2020, Diez et al., 2021, Jungilligens et al., 2021, Goodman et al., 2020, Gninenko et al., 2025, Perez et al., 2021, Lee et al., 2015). However, prior findings remain modest and heterogeneous, potentially reflecting the predominance of DTI with its limitations, low spatial/angular resolution, and variable methodology. Higher fidelity dMRI – with smaller voxels, multi-shell acquisitions, and advanced modelling – offers clearer characterization of structural connectivity alterations in FND. As future UHF systems integrate stronger gradients and improved shimming, 7T may further enhance the precision of tractography and deep-grey segmentation relevant to FND circuitry.

### 3.3 Functional connectivity (fMRI)

fMRI provides a non-invasive measure of large-scale brain dynamics via the BOLD signal, reflecting neuronal activity indirectly through neurovascular coupling. In FND, fMRI has been the most extensively applied advanced neuroimaging modality. A substantial body of work has demonstrated altered resting-state and task-related activity across distributed networks involved in salience processing, emotion regulation, interoception, attention, and motor control, including the salience, default mode, and sensorimotor networks (Perez et al., 2021, Hallett et al., 2022). Despite this progress, most fMRI studies have relied on gradient-echo (GRE) echo-planar imaging (EPI) with millimeter-scale resolution, which limits spatial specificity and favors macrovascular signals. As a result, although network-level connectivity and dynamics can be robustly characterized, such approaches offer limited leverage for directly testing hierarchical or directional hypotheses central to predictive-processing models of FND.

UHF MRI substantially extends the inferential capacity of fMRI by increasing SNR and susceptibility contrast, enabling sub-millimeter spatial resolution and improved sensitivity to microvascular signals. At 7T, voxel sizes can approach cortical layer thickness, permitting layer-specific fMRI when combined with optimized acquisition strategies (see section 2.3.2; (Thomas et al., 2024, Haarsma and Kok, 2025, Finn et al., 2019). Complementary to laminar approaches, gradient-based analyses provide a macroscale framework for probing hierarchical functional organization. The principal cortical connectivity gradient captures a continuous axis from unimodal sensory-motor regions to transmodal (i.e., integrating information across multiple sensory and cognitive systems) default mode areas, reflecting increasing abstraction and integrative processing (Margulies et al., 2016). Sensitivity of this framework to individual differences in emotional vulnerability during naturalistic paradigms suggests relevance for disorders characterized by altered emotional and bodily inference (Ye et al., 2025).

Methodological optimization is particularly important when targeting subcortical and iron-rich structures implicated in motor and cognitive control. High iron content leads to shortened T2* values, imposing constraints on echo time selection and sequence design. Empirical comparisons demonstrate that protocols optimized for cortical fMRI can markedly reduce sensitivity in iron-rich nuclei, whereas short-echo, high-resolution acquisitions improve detectability at 7T (de Hollander et al., 2017). Moreover, multi-echo acquisitions do not universally outperform optimized single-echo protocols when spatial resolution and coverage are controlled, underscoring the need for target-specific protocol design rather than generic assumptions of superiority (Miletić et al., 2020).

Within the proposed biophysical multimodal MRI framework, fMRI occupies a central integrative role, providing a systems-level readout of microstructural properties quantified by qMRI, anatomical pathways characterized by dMRI, and metabolic constraints assessed by MRS approaches. When deployed at UHF and combined with laminar, gradient-based, and protocol-optimized methods, fMRI moves beyond descriptive connectivity mapping to bridge computational theories of hierarchical inference with empirically measurable brain dynamics in FND, enabling mechanistic interrogation of how distributed hierarchical dysfunction contributes to symptom construction.

### 3.4 Neurometabolic Profile (MRS)

Magnetic resonance spectroscopy (MRS) provides a non-invasive window into the biochemical composition of living brain tissue and quantifies metabolites in neurons and glia. Proton MRS (^1^H-MRS) – the clinical standard – resolves metabolite-specific resonance peaks (N-acetyl aspartate, creatine, choline, myo-inositol, glutamate/glutamine, γ-aminobutyric acid) to infer neuronal integrity (NAA), membrane turnover (Cho), glial/osmotic activity (mI), excitatory–inhibitory balance (Glu/Gln and GABA), and cellular energetics (Cr) (Wilson et al., 2019). To date, all MRS research in FND has used single-voxel ^1^H-MRS at 1.5–3T, and no study has applied phosphorus spectroscopy (^31^P-MRS), carbon spectroscopy (^13^C-MRS), sodium spectroscopy, metabolic-flux tracing, spectroscopic imaging (MRSI), or functional MRS paradigms in this population (Lally et al., 2016, Lan et al., 2025, Charney et al., 2024, Simani et al., 2020, Demartini et al., 2019). UHF MRI substantially enhances the capabilities of MRS. Increased magnetic field strength improves SNR ratio and spectral dispersion, enabling better separation of glutamate from glutamine, and improved detection of GABA and glutathione. Advanced 7T approaches – including adiabatic refocusing, semi-localization by adiabatic selective refocusing (semi-LASER) localization – permit higher-resolution mapping of metabolic profiles across cortical and subcortical regions (Wilson et al., 2019). Such improvements are particularly relevant to FND, where pathophysiology likely reflects subtle shifts in excitatory–inhibitory coupling, glial modulation, or arousal-related neurometabolic states rather than overt structural abnormalities.

Several MRS studies indicate neurometabolic abnormalities across limbic, prefrontal, motor, and thalamic circuits in FND. Elevated Glx/Cr has been reported in ACC/medial prefrontal cortex and shown to correlate with alexithymia, anxiety, and motor symptom severity (Demartini et al., 2019). Lower NAA/Cr in dorsomedial prefrontal cortex, ACC, and thalamus, reduced Cho/Cr in ACC, and elevated NAA/Cr in dorsolateral prefrontal cortex have been associated with seizure frequency and deficits in attention and inhibitory control in functional/dissociative seizures (Simani et al., 2020). In pediatric cohorts, reductions in NAA/Cr and mI/Cr in the supplementary motor area and posterior default mode network, as well as lower GABA/Cr in the supplementary motor area, have been observed, with excitatory–inhibitory ratios distinguishing seizure from non-seizure presentations and mI/Cr inversely correlating with autonomic arousal (Charney et al., 2024). Disrupted neurometabolic coupling within the default mode network has also been described using conditional-dependence metabolic network analyses (Lan et al., 2025). Across studies, convergent patterns emerge: reductions in neuronal (NAA/Cr), inhibitory (GABA/Cr), and glial markers (mI/Cr), together with alterations in excitatory metabolites (Glx/Cr), aligning with models of altered excitability, aberrant limbic–motor interactions, impaired top-down control, and dysregulated arousal in FND.

## 4 Translational and clinical outlook

### 4.1 Phenotypic heterogeneity and study design

FND presents with a profound phenotypic heterogeneity, which is evident both across different subtypes – ranging from motor symptoms (weakness, tremor, dystonia, gait disturbances) to seizure-type episodes (functional/dissociative seizures) – and across various disease stages (e.g., active symptoms versus remission). These different clinical presentations may be associated with distinct neuroimaging phenotypes, but based on currently used imaging approaches, the findings are often overlapping. This may reflect shared underlying computational disruptions, but may also indicate insufficient measurement approaches. Building on the premise that a biophysical multimodal MRI framework – particularly at ultra-high field – may reveal microstructural and molecular features inaccessible to conventional imaging, a key challenge is determining how such sensitive approaches map onto clinical presentations (Perez et al., 2021). The goal is to identify the best study designs and methodologies to discover both shared biomarkers (diagnostic and prognostic, pointing to common underlying disruptions) and distinct biomarkers (elucidating the specific biological and molecular differences that drive the observed heterogeneity in symptoms and progression).

This necessary focus on heterogeneity means that neuroimaging studies in FND should move beyond general group comparisons. Study designs require careful stratification by clinical phenotype and symptom state, employing harmonized diagnostic criteria and appropriately matched control groups. Given the high sensitivity of UHF MRI to physiological and state-dependent variability, and the fluctuating nature of FND symptoms, within-subject longitudinal designs (examining individuals across symptomatic and asymptomatic periods) are crucial. Such designs offer maximal power to disentangle trait effects (stable biological features) from state effects (network-level fluctuations tied to symptom expression).

Furthermore, the richness of data derived from this biophysical multimodal MRI framework – spanning high-resolution imaging and connectomics measures – can be leveraged to address this heterogeneity directly. Clustering algorithms, such as the Subtype and Stage Inference (SuStaIn) method, can be applied to identify new, biologically informed subtypes of FND based on their multimodal MRI features (Young et al., 2018). By using sophisticated, data-driven approaches, this research could shed light on specific, objective biomarkers of symptoms and subtypes, and inform personalized treatment strategies, transitioning FND from a largely syndromic diagnosis to one based on specific underlying biological mechanisms.

### 4.2 Technical considerations, tolerability and safety

UHF MRI introduces distinct practical, physiological, and technical considerations compared with conventional clinical field strengths. Transient dizziness can occur at 7T, often during table movement. These effects are primarily attributable to static-field (B_0_) interactions with the vestibular system (Theysohn et al., 2014). A metallic taste has also been reported in a subset of individuals. Practical mitigations include slow table motion and allowing brief pauses if symptoms occur (Özütemiz et al., 2023). Patient tolerability is particularly relevant in FND, as anxiety is common in this population. In 7T clinical workflows, claustrophobia/distress and intolerance to scanner-related sensations are addressed through careful positioning, communication, and protocol tailoring (Özütemiz et al., 2023).

From a safety and sequence-design perspective, SAR constraints become more prominent at UHF. SAR increases roughly with B_0_ (approximately quadratically in practice) and is further influenced by radio frequency (RF) pulse properties and sequence type (e.g., higher SAR for rapid spin-echo/TSE trains vs typically lower SAR for GRE) (Barisano et al., 2019). As a result, protocols often rely on SAR-aware sequence choices and RF strategies, including methods that shape transmission profiles while minimizing deposited RF energy (Balchandani and Naidich 2015). Dielectric pads can be used to modify the local B_1_^+^ distribution and, in some configurations, may reduce required input power for a target region – though their impact on SAR estimation requires caution and appropriate safety considerations (Fagan et al., 2021). Parallel transmission is important for addressing UHF RF-field non-uniformity and SAR constraints (Padormo et al., 2016). While the increased B_0_ improves SNR and enables higher spatial resolution, it also amplifies field-related artefact mechanisms – most prominently B_0_ and B_1_^+^ inhomogeneity, EPI-related distortion and signal non-uniformity, and susceptibility-related effects – requiring dedicated hardware and sequence optimization. These effects, and corresponding mitigation strategies, are summarized in Supplementary Box 1.

Importantly, several limitations of UHF MRI are practical and translational rather than purely physical. Compared with 3T, 7T remains less widely available and is more sensitive to site-specific factors (scanner platform, RF coil configuration, shimming/calibration workflows, and sequence variants), which can complicate large-scale multi-site acquisition and harmonization (Clarke et al., 2020). Multi-center “traveling-head” studies (i.e., the same participants scanned across multiple sites) show that quantitative protocols at 7T can achieve high reproducibility across sites, while also revealing systematic differences attributable to calibration and RF coil/B_1_-related procedures (Voelker et al., 2021). Disease-focused network efforts further support the feasibility of standardized 7T protocols across multiple sites, but also underscore the need for careful protocol governance and harmonization infrastructure (Düzel et al., 2019). Consequently, while UHF MRI can provide unique biological specificity, standard field MRI is more scalable for large multi-center cohorts. A pragmatic approach is therefore to use 7T to refine hypotheses/biomarkers and subsequently test generalizability in larger 3T samples.

Overall, the benefits and limitations of UHF MRI must be explicitly balanced during study planning. Although UHF MRI offers additional opportunities for high-resolution, biophysically informed imaging, it is not universally optimal. Careful consideration of patient tolerability, safety constraints, and technical trade-offs is essential.

### 4.3 Integration and multimodal roadmap

Integration of UHF and biophysical multimodal MRI in FND will, in the short term, consist of feasibility and reliability studies in small cohorts that establish test–retest reliability of 7T measures such as LC neuromelanin contrast, neocortical laminar activity, and basal-ganglia QSM (Weiskopf et al., 2021). In the medium term, multimodal integration of qMRI, dMRI, fMRI, and MRS acquired within the same individual is central to characterizing microstructure–function-metabolism coupling. Crucially, this multimodal approach will move beyond mass univariate analysis – which examines how each neuroimaging feature in isolation relates to pathology or symptoms – to multivariate approaches. These multivariate models, often using data-driven fusion methods like canonical correlation analysis or manifold embedding, will allow for the synergistic combination of multiple neuroimaging features that better explain the observed symptoms and pathology. Additionally, multimodal integration can also clarify associations between different neuroimaging-derived phenotypes, for example, how structural alterations might lead to observed functional changes (Smith et al., 2015). In the longer term, clinically validated 7T biomarkers could support personalized therapy; for example, laminar SMA or rTPJ activity may help guide non-invasive brain stimulation approaches such as transcranial magnetic stimulation (TMS) or transcranial direct current stimulation (tDCS) (Fox et al., 2014). Achieving this trajectory will require large-scale harmonization efforts similar to the Alzheimer’s Disease Neuroimaging Initiative (ADNI, https://adni.loni.usc.edu) or ENIGMA (Enhancing Neuroimaging Genetics by Meta-Analysis, http://enigma.ini.usc.edu). Open sharing of high-field data sets and preprocessing code will accelerate reproducibility and methodological convergence.

Looking ahead, emerging UHF experimental platforms illustrate the longer-term horizon for translational imaging. Recent work demonstrates that human cortical circuitry can be resolved at laminar and columnar scales. Now, using ultra-strong gradients and specialized RF engineering, sub-0.5 mm fMRI is becoming feasible, enabling near-mesoscopic readouts of cortical processing (Feinberg et al., 2023). Although such hardware is not currently deployable in clinical or multi-center research settings, these advances define the physiological scales that future UHF scanners may reach and underscore the value of designing FND imaging paradigms that will remain compatible with forthcoming high-resolution fMRI technologies.

### 4.4 Conceptual integration

UHF MRI will not “explain away” FND but can further connect symptom expression to quantifiable brain network mechanisms. By measuring excitation–inhibition balance (MRS), microstructural integrity (qMRI), laminar information flow (fMRI), and tract topology (dMRI) within the same individuals, our understanding could move from correlative network dysfunction towards mechanistic inference. Integrating these findings with behavioral and physiological markers – e.g., electroencephalography, eye-tracking, pupillometry, electromyography, and galvanic skin response – will allow cross-validation of circuit models. The rich, high-resolution data obtained from the integration of all these imaging modalities are particularly powerful for informing personalized treatment strategies, especially those involving neuromodulation. Specifically, a connectomics approach can be exploited to infer the most effective brain hubs for intervention. Ultimately, integration of UHF MRI with non-invasive neuromodulation in FND and invasive neuromodulation in symptom-overlapping disorders may support a transdiagnostic approach (Husain, 2017), allowing causal testing of symptom-relevant brain circuits and paving the way for targeted circuit therapeutics (Siddiqi et al., 2024).

## 5 Conclusion

UHF MRI offers a window on brain structure, function, and chemistry, extending beyond the spatial and contrast constraints of conventional clinical imaging. In this review, we outline a biophysical multimodal MRI framework that leverages complementary measurements of microstructure, connectivity, dynamics, and metabolism to interrogate the mechanisms underlying FND. By enabling more precise characterization of subnuclear architecture, laminar- and network-level dynamics, and neurometabolic states, UHF MRI can uncover features of symptom construction that remain invisible at standard field strengths, and distinguish disease-relevant pathways from comorbid or compensatory processes. Beyond advancing FND research, this framework may position FND as a model condition for investigating reversible, network-based brain dysfunction at the interface of neurology and psychiatry.

## Declaration of generative AI and AI-assisted technologies in the manuscript preparation process

During the preparation of this work, the authors used generative AI (Large Language models, LLMs) to assist in literature exploration and refine wording. After using this tool, the authors reviewed and edited the contents as needed and take full responsibility for the content of the published article.

## CRediT authorship contribution statement

**Sverre Myren:** Conceptualization, Investigation, Validation, Visualization, Writing – original draft, Writing – review & editing. **Johannes Jungilligens:** Conceptualization, Investigation, Validation, Visualization, Writing – original draft, Writing – review & editing. **Ibai Diez:** Investigation, Validation, Writing – review & editing. **Erlend Bøen:** Investigation, Validation, Writing – review & editing. **Torbjørn Elvsåshagen:** Investigation, Validation, Writing – review & editing. **Birte Forstmann:** Investigation, Validation, Writing – review & editing. **Maryam Ziaei:** Investigation, Validation, Writing – review & editing. **Thanh P. Doan:** Conceptualization, Investigation, Validation, Visualization, Writing – original draft, Writing – review & editing.

## Declaration of competing interest

The authors declare the following financial interests/personal relationships which may be considered as potential competing interests: E.B. has received lecture honoraria from Lundbeck. T.E. has received honoraria from Cumulus Neuroscience Ltd. and Sumitomo Pharma America. The remaining authors have no conflicts of interest to declare.

## Data Availability

No data was used for the research described in the article.

## Appendix A Supplementary data

Supplementary Box 1 Technical considerations and artefact mitigation at 7T.

## References

[b0005] Atmaca M., Aydin A., Tezcan E., Poyraz A.K., Kara B. (2006). Volumetric investigation of brain regions in patients with conversion disorder. Prog. Neuropsychopharmacol. Biol. Psychiatry.

[b0015] Aybek S., Perez D.L. (2022). Diagnosis and management of functional neurological disorder. BMJ.

[b0020] Aybek S., Nicholson T.R., Zelaya F., O’Daly O.G., Craig T.J., David A.S., Kanaan R.A. (2014). Neural correlates of recall of life events in conversion disorder. JAMA Psychiat..

[b0010] Aybek S., Nicholson T.R., O'Daly O., Zelaya F., Kanaan R.A., David A.S. (2015). Emotion-motion interactions in conversion disorder: an FMRI study. PLoS One.

[b0025] Baek K., Doñamayor N., Morris L.S., Strelchuk D., Mitchell S., Mikheenko Y., Yeoh S.Y., Phillips W., Zandi M., Jenaway A., Walsh C., Voon V. (2017). Impaired awareness of motor intention in functional neurological disorder: implications for voluntary and functional movement. Psychol. Med..

[b0030] Balchandani P., Naidich T.P. (2015). Ultra-high-field MR neuroimaging. AJNR Am. J. Neuroradiol..

[b0035] Barisano G., Sepehrband F., Ma S., Jann K., Cabeen R., Wang D.J., Toga A.W., Law M. (2019). Clinical 7 T MRI: are we there yet? a review about magnetic resonance imaging at ultra-high field. Br. J. Radiol..

[b0040] Basser P.J., Mattiello J., LeBihan D. (1994). MR diffusion tensor spectroscopy and imaging. Biophys. J..

[b0045] Bazin P.-L., Groot J.M., Miletic S., Groenewegen L., Trutti A.C., Mulder M.J., Forstmann B.U., Alkemade A. (2025). Automated parcellation and atlasing of the human subcortex with ultra-high resolution quantitative MRI. Imaging Neurosci..

[b0050] Bègue I., Adams C., Stone J., Perez D.L. (2019). Structural alterations in functional neurological disorder and related conditions: a software and hardware problem?. Neuroimage Clin.

[b0055] Behbehani M.M. (1995). Functional characteristics of the midbrain periaqueductal gray. Prog. Neurobiol..

[b0060] Bender B., Mänz C., Korn A., Nägele T., Klose U. (2011). Optimized 3D magnetization-prepared rapid acquisition of gradient echo: identification of thalamus substructures at 3T. AJNR Am. J. Neuroradiol..

[b0065] Berron D., Vieweg P., Hochkeppler A., Pluta J.B., Ding S.L., Maass A., Luther A., Xie L., Das S.R., Wolk D.A., Wolbers T., Yushkevich P.A., Düzel E., Wisse L.E.M. (2017). A protocol for manual segmentation of medial temporal lobe subregions in 7 Tesla MRI. Neuroimage Clin.

[b0070] Betts M.J., Kirilina E., Otaduy M.C.G., Ivanov D., Acosta-Cabronero J., Callaghan M.F., Lambert C., Cardenas-Blanco A., Pine K., Passamonti L., Loane C., Keuken M.C., Trujillo P., Lüsebrink F., Mattern H., Liu K.Y., Priovoulos N., Fliessbach K., Dahl M.J., Maaß A., Madelung C.F., Meder D., Ehrenberg A.J., Speck O., Weiskopf N., Dolan R., Inglis B., Tosun D., Morawski M., Zucca F.A., Siebner H.R., Mather M., Uludag K., Heinsen H., Poser B.A., Howard R., Zecca L., Rowe J.B., Grinberg L.T., Jacobs H.I.L., Düzel E., Hämmerer D. (2019). Locus coeruleus imaging as a biomarker for noradrenergic dysfunction in neurodegenerative diseases. Brain.

[b0075] Bianciardi M., Strong C., Toschi N., Edlow B.L., Fischl B., Brown E.N., Rosen B.R., Wald L.L. (2018). A probabilistic template of human mesopontine tegmental nuclei from in vivo 7T MRI. Neuroimage.

[b0080] Blakemore R.L., Sinanaj I., Galli S., Aybek S., Vuilleumier P. (2016). Aversive stimuli exacerbate defensive motor behaviour in motor conversion disorder. Neuropsychologia.

[b0085] Bouhrara M., Reiter D.A., Bergeron C.M., Zukley L.M., Ferrucci L., Resnick S.M., Spencer R.G. (2018). Evidence of demyelination in mild cognitive impairment and dementia using a direct and specific magnetic resonance imaging measure of myelin content. Alzheimers Dement..

[b0090] Brammerloh M., Kirilina E., Alkemade A., Bazin P.L., Jantzen C., Jäger C., Herrler A., Pine K.J., Gowland P.A., Morawski M., Forstmann B.U., Weiskopf N. (2022). Swallow Tail Sign: Revisited. Radiology.

[b0095] Brown S.S.G., Rutland J.W., Verma G., Feldman R.E., Alper J., Schneider M., Delman B.N., Murrough J.M., Balchandani P. (2019). Structural MRI at 7T reveals amygdala nuclei and hippocampal subfield volumetric association with major depressive disorder symptom severity. Sci. Rep..

[b0100] Bühler J., Weber S., Loukas S., Walther S., Aybek S. (2024). Non-invasive neuromodulation of the right temporoparietal junction using theta-burst stimulation in functional neurological disorder. BMJ Neurology Open.

[b0105] Cacciola A., Bertino S., Basile G.A., Di Mauro D., Calamuneri A., Chillemi G., Duca A., Bruschetta D., Flace P., Favaloro A., Calabrò R.S., Anastasi G., Milardi D. (2019). Mapping the structural connectivity between the periaqueductal gray and the cerebellum in humans. Brain Struct. Funct..

[b0110] Charney M., Foster S., Shukla V., Zhao W., Jiang S.H., Kozlowska K., Lin A. (2024). Neurometabolic alterations in children and adolescents with functional neurological disorder. Neuroimage Clin.

[b0115] Clarke W.T., Mougin O., Driver I.D., Rua C., Morgan A.T., Asghar M., Clare S., Francis S., Wise R.G., Rodgers C.T., Carpenter A., Muir K., Bowtell R. (2020). Multi-site harmonization of 7 tesla MRI neuroimaging protocols. Neuroimage.

[b0120] Dave A., Ye S., Bätz L.R., Lan X., Jacobs H.I.L., Ziaei M. (2025). Age-related increase in locus ceruleus activity and connectivity with the prefrontal cortex during ambiguity processing. J. Neurosci..

[b0125] de Hollander G., Keuken M.C., van der Zwaag W., Forstmann B.U., Trampel R. (2017). Comparing functional MRI protocols for small, iron-rich basal ganglia nuclei such as the subthalamic nucleus at 7 T and 3 T. Hum. Brain Mapp..

[b0130] Demartini B., Gambini O., Uggetti C., Cariati M., Cadioli M., Goeta D., Marceglia S., Ferrucci R., Priori A. (2019). Limbic neurochemical changes in patients with functional motor symptoms. Neurology.

[b0135] Derix J., Yang S., Lüsebrink F., Fiederer L.D., Schulze-Bonhage A., Aertsen A., Speck O., Ball T. (2014). Visualization of the amygdalo-hippocampal border and its structural variability by 7T and 3T magnetic resonance imaging. Hum. Brain Mapp..

[b0140] *Diagnostic and Statistical Manual of Mental Disorders, Fifth Edition, Text Revision (DSM-5-TR)*. 2022. (American Psychiatric Association).

[b0145] Diez I., Ortiz-Terán L., Williams B., Jalilianhasanpour R., Ospina J.P., Dickerson B.C., Keshavan M.S., LaFrance W.C., Sepulcre J., Perez D.L. (2019). Corticolimbic fast-tracking: enhanced multimodal integration in functional neurological disorder. J. Neurol. Neurosurg. Psychiatry.

[b0150] Diez I., Williams B., Kubicki M.R., Makris N., Perez D.L. (2021). Reduced limbic microstructural integrity in functional neurological disorder. Psychol. Med..

[b0530] Dorocic P., Iskra D.F., Xuan Y., Johansson Y., Pozzi L., Silberberg G., Carlén M., Meletis K. (2014). A whole-brain atlas of inputs to serotonergic neurons of the dorsal and median raphe nuclei. Neuron.

[b0155] Drane D.L., Fani N., Hallett M., Khalsa S.S., Perez D.L., Roberts N.A. (2020). A framework for understanding the pathophysiology of functional neurological disorder. CNS Spectr..

[b0160] Drori E., Cohen L., Arkadir D., Yahalom G., Mezer A.A. (2025). Multiparametric quantitative MRI uncovers putamen microstructural changes in Parkinson's disease. NPJ Parkinsons Dis.

[b0165] Düzel E., Acosta-Cabronero J., Berron D., Jan Biessels G., Björkman-Burtscher I., Bottlaender M., Bowtell R., Buchem M.v., Cardenas-Blanco A., Boumezbeur F., Chan D., Clare S., Costagli M., de Rochefort L., Fillmer A., Gowland P., Hansson O., Hendrikse J., Kraff O., Ladd M.E., Ronen I., Petersen E., Rowe J.B., Siebner H., Stoecker T., Straub S., Tosetti M., Uludag K., Vignaud A., Zwanenburg J., Speck O. (2019). European Ultrahigh-Field Imaging Network for Neurodegenerative Diseases (EUFIND). Alzheimer's & Dementia: Diagnosis, Assessment & Disease Monitoring.

[b0170] Edwards M.J., Adams R.A., Brown H., Pareés I., Friston K.J. (2012). A Bayesian account of 'hysteria. Brain.

[b0175] Eichenbaum H. (2017). The role of the hippocampus in navigation is memory. J. Neurophysiol..

[b0180] Elkommos S., Martin-Lopez D., Koreki A., Jolliffe C., Kandasamy R., Mula M., Critchley H.D., Edwards M.J., Garfinkel S., Richardson M.P., Yogarajah M. (2023). Changes in the heartbeat-evoked potential are associated with functional seizures. J. Neurol. Neurosurg. Psychiatry.

[b0185] Ezra M., Faull O.K., Jbabdi S.a., Pattinson K.T. (2015). Connectivity-based segmentation of the periaqueductal gray matter in human with brainstem optimized diffusion MRI. Hum. Brain Mapp..

[b0190] Fabio M., Rachele P., Sara L., Dante M., Cristina S., Giorgio A. (2022). Disconnection from prediction: a systematic review on the role of right temporoparietal junction in aberrant predictive processing. Neurosci. Biobehav. Rev..

[b0195] Fagan A.J., Bitz A.K., Björkman-Burtscher I.M., Collins C.M., Kimbrell V., Raaijmakers A.J.E. (2021). 7T MR Safety. J. Magn. Reson. Imaging.

[b0200] Fartaria M.J., OʼBrien K., Şorega A., Bonnier G., Roche A., Falkovskiy P., Krueger G., Kober T., Bach Cuadra M., Granziera C. (2017). An ultra-high field study of cerebellar pathology in early relapsing-remitting multiple sclerosis using MP2RAGE. Invest. Radiol..

[b0205] Faull O.K., Jenkinson M., Clare S., Pattinson K.T. (2015). Functional subdivision of the human periaqueductal grey in respiratory control using 7 tesla fMRI. Neuroimage.

[b0210] Feinberg D.A., Beckett A.J.S., Vu A.T., Stockmann J., Huber L., Ma S., Ahn S., Setsompop K., Cao X., Park S., Liu C., Wald L.L., Polimeni J.R., Mareyam A., Gruber B., Stirnberg R., Liao C., Yacoub E., Davids M., Bell P., Rummert E., Koehler M., Potthast A., Gonzalez-Insua I., Stocker S., Gunamony S., Dietz P. (2023). Next-generation MRI scanner designed for ultra-high-resolution human brain imaging at 7 Tesla. Nat. Methods.

[b0215] Finn E.S., Huber L., Jangraw D.C., Molfese P.J., Bandettini P.A. (2019). Layer-dependent activity in human prefrontal cortex during working memory. Nat. Neurosci..

[b0220] Flasbeck V., Jungilligens J., Lemke I., Beckers J., Öztürk H., Wellmer J., Seliger C., Juckel G., Popkirov S. (2024). Heartbeat evoked potentials and autonomic arousal during dissociative seizures: insights from electrophysiology and neuroimaging. BMJ Neurol. Open.

[b0225] Fox M.D., Buckner R.L., Hesheng Liu M., Chakravarty M., Lozano A.M., Pascual-Leone A. (2014). Resting-state networks link invasive and noninvasive brain stimulation across diverse psychiatric and neurological diseases. Proc. Natl. Acad. Sci..

[b0230] Gallichan D. (2018). Diffusion MRI of the human brain at ultra-high field (UHF): a review. Neuroimage.

[b0235] Gninenko N., Müller E., Aybek S. (2025). Reduced microstructural white matter integrity is associated with the severity of physical symptoms in functional neurological disorder. Neuroimage Clin..

[b0240] Goldstein L.H., Mellers J.D.C. (2006). Ictal symptoms of anxiety, avoidance behaviour, and dissociation in patients with dissociative seizures. J. Neurol. Neurosurg. Psychiatry.

[b0245] Goodman A.M., Allendorfer J.B., Blum A.S., Bolding M.S., Correia S., Ver Hoef L.W., Gaston T.E., Grayson L.E., Kraguljac N.V., Lahti A.C., Martin A.N., Monroe W.S., Philip N.S., Tocco K., Vogel V., LaFrance W.C., Szaflarski J.P. (2020). White matter and neurite morphology differ in psychogenic nonepileptic seizures. Ann. Clin. Transl. Neurol..

[b0250] Grodd W., Kumar V.J., Schüz A., Lindig T., Scheffler K. (2020). The anterior and medial thalamic nuclei and the human limbic system: tracing the structural connectivity using diffusion-weighted imaging. Sci. Rep..

[b0305] Haarsma J., Kok P., Khalsa S., Powers A. (2025). *Perceptual Dysregulation in Psychiatric Nosology*.

[b0255] Hallett M. (2024). Functional Neurologic Disorder, La Lésion Dynamique: 2024 Wartenberg Lecture. Neurology.

[b0260] Hallett M., Aybek S., Dworetzky B.A., McWhirter L., Staab J.P., Stone J. (2022). Functional neurological disorder: new subtypes and shared mechanisms. Lancet Neurol..

[b0265] Hassa T., Sebastian A., Liepert J., Weiller C., Schmidt R., Tüscher O. (2017). Symptom-specific amygdala hyperactivity modulates motor control network in conversion disorder. Neuroimage Clin..

[b0270] Hassan J., Taib S., Yrondi A. (2024). Structural and functional changes associated with functional/dissociative seizures: a review of the literature. Epilepsy Behav..

[b0275] Heij J., van der Zwaag W., Knapen T., Caan M.W.A., Forstman B., Veltman D.J., van Wingen G., Aghajani M. (2024). Quantitative MRI at 7-Tesla reveals novel frontocortical myeloarchitecture anomalies in major depressive disorder. Transl. Psychiatry.

[b0280] Hernando K.A., Szaflarski J.P., Ver Hoef L.W., Lee S., Allendorfer J.B. (2015). Uncinate fasciculus connectivity in patients with psychogenic nonepileptic seizures: a preliminary diffusion tensor tractography study. Epilepsy Behav..

[b0285] Huang S.Y., Witzel T., Keil B., Scholz A., Davids M., Dietz P., Rummert E., Ramb R., Kirsch J.E., Yendiki A., Fan Q., Tian Q., Ramos-Llordén G., Lee H.H., Nummenmaa A., Bilgic B., Setsompop K., Wang F., Avram A.V., Komlosh M., Benjamini D., Magdoom K.N., Pathak S., Schneider W., Novikov D.S., Fieremans E., Tounekti S., Mekkaoui C., Augustinack J., Berger D., Shapson-Coe A., Lichtman J., Basser P.J., Wald L.L., Rosen B.R. (2021). Connectome 2.0: developing the next-generation ultra-high gradient strength human MRI scanner for bridging studies of the micro-, meso- and macro-connectome. Neuroimage.

[b0290] Huber L., Handwerker D.A., Jangraw D.C., Chen G., Hall A., Stüber C., Gonzalez-Castillo J., Ivanov D., Marrett S., Guidi M., Goense J., Poser B.A., Bandettini P.A. (2017). High-resolution CBV-fMRI allows mapping of laminar activity and connectivity of cortical input and output in human M1. Neuron.

[b0295] Husain M. (2017). Transdiagnostic neurology: neuropsychiatric symptoms in neurodegenerative diseases. Brain.

[b0300] Huys A.M.L., Haggard P., Bhatia K.P., Edwards M.J. (2021). Misdirected attentional focus in functional tremor. Brain.

[b0310] Jacobs B.L., Azmitia E.C. (1992). Structure and function of the brain serotonin system. Physiol. Rev..

[b0315] Janet R., Costes N., Mérida I., Derrington E., Dreher J.C. (2023). Relationships between serotonin availability and frontolimbic response to fearful and threatening faces. Sci. Rep..

[b0320] Jeurissen B., Leemans A., Tournier J.D., Jones D.K., Sijbers J. (2013). Investigating the prevalence of complex fiber configurations in white matter tissue with diffusion magnetic resonance imaging. Hum. Brain Mapp..

[b0325] Jungilligens J., Perez D.L. (2025). Predictive processing and the pathophysiology of functional neurological disorder. Curr. Top. Behav. Neurosci..

[b0335] Jungilligens J., Wellmer J., Kowoll A., Schlegel U., Axmacher N., Popkirov S. (2021). Microstructural integrity of affective neurocircuitry in patients with dissociative seizures is associated with emotional task performance, illness severity and trauma history. Seizure.

[b0330] Jungilligens J., Popkirov S., Perez D.L., Diez I. (2022). Linking gene expression patterns and brain morphometry to trauma and symptom severity in patients with functional seizures. Psychiatry Res. Neuroimaging.

[b0340] Jungilligens J., Paredes-Echeverri S., Popkirov S., Barrett L.F., Perez D.L. (2022). A new science of emotion: implications for functional neurological disorder. Brain.

[b0345] Kanai R., Komura Y., Shipp S., Friston K. (2015).

[b0350] Kerchner G.A., Deutsch G.K., Zeineh M., Dougherty R.F., Saranathan M., Rutt B.K. (2012). Hippocampal CA1 apical neuropil atrophy and memory performance in Alzheimer's disease. Neuroimage.

[b0355] Keuken M.C., Forstmann B.U. (2015). A probabilistic atlas of the basal ganglia using 7 T MRI. Data Brief.

[b0360] Koopmans P.J., Barth M., Orzada S., Norris D.G. (2011). Multi-echo fMRI of the cortical laminae in humans at 7T. Neuroimage.

[b0365] Krall S.C., Rottschy C., Oberwelland E., Bzdok D., Fox P.T., Eickhoff S.B., Fink G.R., Konrad K. (2015). The role of the right temporoparietal junction in attention and social interaction as revealed by ALE meta-analysis. Brain Struct. Funct..

[b0370] Kranick S.M., Moore J.W., Yusuf N., Martinez V.T., LaFaver K., Edwards M.J., Mehta A.R., Collins P., Harrison N.A., Haggard P., Hallett M., Voon V. (2013). Action-effect binding is decreased in motor conversion disorder: implications for sense of agency. Mov. Disord..

[b0375] Labate A., Cerasa A., Mula M., Mumoli L., Gioia M.C., Aguglia U., Quattrone A., Gambardella A. (2012). Neuroanatomic correlates of psychogenic nonepileptic seizures: a cortical thickness and VBM study. Epilepsia.

[b0380] Lally N., An L., Banerjee D., Niciu M.J., Luckenbaugh D.A., Richards E.M., Roiser J.P., Shen J., Zarate C.A., Nugent A.C. (2016). Reliability of 7T (1) H-MRS measured human prefrontal cortex glutamate, glutamine, and glutathione signals using an adapted echo time optimized PRESS sequence: a between- and within-sessions investigation. J. Magn. Reson. Imaging.

[b0385] Lan Z., Foster S., Charney M., van Grinsven M., Breedlove K., Kozlowska K., Lin A. (2025). Neurometabolic network (NMetNet) for functional neurological disorder in children and adolescents. Neuroimage Clin..

[b0390] LeDoux J.E. (2000). Emotion circuits in the brain. Annu. Rev. Neurosci..

[b0395] Lee S., Allendorfer J.B., Gaston T.E., Griffis J.C., Hernando K.A., Knowlton R.C., Szaflarski J.P., Ver Hoef L.W. (2015). White matter diffusion abnormalities in patients with psychogenic non-epileptic seizures. Brain Res..

[b0400] Li R., Liu K., Ma X., Li Z., Duan X., An D., Gong Q., Zhou D., Chen H. (2015). Altered functional connectivity patterns of the insular subregions in psychogenic nonepileptic seizures. Brain Topogr..

[b0405] Lin D., Castro P., Edwards A., Sekar A., Edwards M.J., Coebergh J., Bronstein A.M., Kaski D. (2020). Dissociated motor learning and de-adaptation in patients with functional gait disorders. Brain.

[b0410] Machen B., Miller S.N., Xin A., Lampert C., Assaf L., Tucker J., Herrell S., Pereira F., Loewinger G., Beas S. (2026). The encoding of interoceptive-based predictions by the paraventricular nucleus of the thalamus D2R+ neurons. iScience.

[b0415] Margulies D.S., Ghosh S.S., Goulas A., Falkiewicz M., Huntenburg J.M., Langs G., Bezgin G., Eickhoff S.B., Castellanos F.X., Petrides M., Jefferies E., Smallwood J. (2016). Situating the default-mode network along a principal gradient of macroscale cortical organization. PNAS.

[b0420] Maurer C.W., LaFaver K., Ameli R., Epstein S.A., Hallett M., Horovitz S.G. (2016). Impaired self-agency in functional movement disorders: a resting-state fMRI study. Neurology.

[b0425] Maurer C.W., LaFaver K., Limachia G.S., Capitan G., Ameli R., Sinclair S., Epstein S.A., Hallett M., Horovitz S.G. (2018). Gray matter differences in patients with functional movement disorders. Neurology.

[b0430] McSweeney M., Reuber M., Levita L. (2017). Neuroimaging studies in patients with psychogenic non-epileptic seizures: a systematic meta-review. Neuroimage Clin.

[b0435] Miletić S., Bazin P.L., Weiskopf N., van der Zwaag W., Forstmann B.U., Trampel R. (2020). fMRI protocol optimization for simultaneously studying small subcortical and cortical areas at 7T. Neuroimage.

[b0440] Millman L.S.M., Short E., Stanton B., Winston J.S., Nicholson T.R., Mehta M.A., Aats Reinders M.J., Edwards L.H., Goldstein A.S., David M., Hotopf T.C., Pick S. (2023). Interoception in functional motor symptoms and functional seizures: Preliminary evidence of intact accuracy alongside reduced insight and altered sensibility. Behav. Res. Ther..

[b0445] Mueller S.G., Garga N., Garcia P., Rossi S., Vu A., Neylan T., Laxer K.D. (2024). The imprint of dissociative seizures on the brain. Neuroimage Clin..

[b0450] Nahab F.B., Kundu P., Maurer C., Shen Q., Hallett M. (2017). Impaired sense of agency in functional movement disorders: an fMRI study. PLoS One.

[b0455] Nicholson T.R., Aybek S., Kempton M.J., Daly E.M., Murphy D.G., David A.S., Kanaan R.A. (2014). A structural MRI study of motor conversion disorder: evidence of reduction in thalamic volume. J. Neurol. Neurosurg. & Psychiatry.

[b0460] Ospina J.P., Jalilianhasanpour R., Perez D.L. (2019). The role of the anterior and midcingulate cortex in the neurobiology of functional neurologic disorder. Handb. Clin. Neurol..

[b0465] Ostuzzi G., Geroin C., Gastaldon C., Tedeschi F., Clesi F.M., Trevisan G., Bidello G., Vita G., Marcuzzo E., Sandri A., Romito L.M., Eleopra R., Tesolin L., Franch I., Zappia M., Nicoletti A., Demartini B., Nisticò V., Modugno N., Olivola E., Pilotto A., Padovani A., Defazio G., Ercoli T., Petracca M., De Micco R., Dallocchio C., Esposito M., Erro R., Del Prete E., Amaddeo F., Barbui C., Tinazzi M. (2025). Characterising alexithymia in individuals with functional motor disorders: a cross-sectional analysis of the Italian Registry of Functional Motor Disorders. J. Neurol. Neurosurg. Psychiatry.

[b0820] Özütemiz C., White M., Elvendahl W., Eryaman Y., Marjańska M., Metzger G.J., Patriat R., Kulesa J., Harel N., Watanabe Y., Grant A., Genovese G., Cayci Z. (2023). Use of a commercial 7-T MRI scanner for clinical brain imaging: indications, protocols, challenges, and solutions-a single-center experience. AJR Am. J. Roentgenol..

[b0470] Padormo F., Beqiri A., Hajnal J.V., Malik S.J. (2016). Parallel transmission for ultrahigh-field imaging. NMR Biomed..

[b0475] Paredes-Echeverri S., Maggio J., Bègue I., Pick S., Nicholson T.R., Perez D.L. (2022). Autonomic, endocrine, and inflammation profiles in functional neurological disorder: a systematic review and meta-analysis. J. Neuropsychiatry Clin. Neurosci..

[b0480] Pareés I., Kassavetis P., Saifee T.A., Sadnicka A., Bhatia K.P., Fotopoulou A., Edwards M.J. (2012). Jumping to conclusions' bias in functional movement disorders. J. Neurol. Neurosurg. Psychiatry.

[b0485] Pareés I., Saifee T.A., Kassavetis P., Kojovic M., Rubio-Agusti I., Rothwell J.C., Bhatia K.P., Edwards M.J. (2012). Believing is perceiving: mismatch between self-report and actigraphy in psychogenic tremor. Brain.

[b0500] Perez D.L., Williams B., Matin N., LaFrance W.C., Costumero-Ramos V., Fricchione G.L., Sepulcre J., Keshavan M.S., Dickerson B.C. (2017). Corticolimbic structural alterations linked to health status and trait anxiety in functional neurological disorder. J. Neurol. Neurosurg. Psychiatry.

[b0490] Perez D.L., Matin N., Williams B., Tanev K., Makris N., LaFrance W.C., Dickerson B.C. (2018). Cortical thickness alterations linked to somatoform and psychological dissociation in functional neurological disorders. Hum. Brain Mapp..

[b0495] Perez D.L., Nicholson T.R., Asadi-Pooya A.A., Bègue I., Butler M., Carson A.J., David A.S., Deeley Q., Diez I., Edwards M.J., Espay A.J., Gelauff J.M., Hallett M., Horovitz S.G., Jungilligens J., Kanaan R.A.A., Tijssen M.A.J., Kozlowska K., LaFaver K., LaFrance W.C., Lidstone S.C., Marapin R.S., Maurer C.W., Modirrousta M., Reinders A., Sojka P., Staab J.P., Stone J., Szaflarski J.P., Aybek S. (2021). Neuroimaging in Functional Neurological Disorder: State of the Field and Research Agenda. Neuroimage Clin.

[b0505] Pessoa L. (2017). A network model of the emotional brain. Trends Cogn. Sci..

[b0510] Pessoa L., Adolphs R. (2010). Emotion processing and the amygdala: from a 'low road' to 'many roads' of evaluating biological significance. Nat. Rev. Neurosci..

[b0515] Planche V., Su J.H., Mournet S., Saranathan M., Dousset V., Han M., Rutt B.K., Tourdias T. (2020). White-matter-nulled MPRAGE at 7T reveals thalamic lesions and atrophy of specific thalamic nuclei in multiple sclerosis. Mult. Scler..

[b0520] Poe G.R., Foote S., Eschenko O., Johansen J.P., Bouret S., Aston-Jones G., Harley C.W., Manahan-Vaughan D., Weinshenker D., Valentino R., Berridge C., Chandler D.J., Waterhouse B., Sara S.J. (2020). Locus coeruleus: a new look at the blue spot. Nat. Rev. Neurosci..

[b0525] Pohmann R., Speck O., Scheffler K. (2016). Signal-to-noise ratio and MR tissue parameters in human brain imaging at 3, 7, and 9.4 tesla using current receive coil arrays. Magn. Reson. Med..

[b0540] Priovoulos N., Jacobs H.I.L., Ivanov D., Uludağ K., Verhey F.R.J., Poser B.A. (2018). High-resolution in vivo imaging of human locus coeruleus by magnetization transfer MRI at 3T and 7T. Neuroimage.

[b0535] Priovoulos N., Andersen M., Dumoulin S.O., Boer V.O., van der Zwaag W. (2023). High-resolution motion-corrected 7.0-T MRI to derive morphologic measures from the human cerebellum in vivo. Radiology.

[b0545] Rikhye R.V., Wimmer R.D., Halassa M.M. (2018). Toward an integrative theory of thalamic function. Annu. Rev. Neurosci..

[b0550] Rubin R.D., Watson P.D., Duff M.C., Cohen N.J. (2014). The role of the hippocampus in flexible cognition and social behavior. Front. Hum. Neurosci..

[b0645] Saalmann Y.B., Pinsk M.A., Wang L., Li X., Kastner S. (2012). The pulvinar regulates information transmission between cortical areas based on attention demands. Science.

[b0555] Sadnicka A., Daum C., Meppelink A.M., Manohar S., Edwards M. (2020). Reduced drift rate: a biomarker of impaired information processing in functional movement disorders. Brain.

[b0560] Sasaki M., Shibata E., Tohyama K., Takahashi J., Otsuka K., Tsuchiya K., Takahashi S., Ehara S., Terayama Y., Sakai A. (2006). Neuromelanin magnetic resonance imaging of locus ceruleus and substantia nigra in Parkinson's disease. Neuroreport.

[b0565] Sasikumar S., Strafella A.P. (2021). The neuroimaging evidence of brain abnormalities in functional movement disorders. Brain.

[b0570] Saygin Z.M., Kliemann D., Iglesias J.E., van der Kouwe A.J.W., Boyd E., Reuter M., Stevens A., Van Leemput K., McKee A., Frosch M.P., Fischl B., Augustinack J.C. (2017). High-resolution magnetic resonance imaging reveals nuclei of the human amygdala: manual segmentation to automatic atlas. Neuroimage.

[b0575] Segobin S., Haast R.A.M., Kumar V.J., Lella A., Alkemade A., Bach Cuadra M., Barbeau E.J., Felician O., Pergola G., Pitel A.L., Saranathan M., Tourdias T., Hornberger M. (2024). A roadmap towards standardized neuroimaging approaches for human thalamic nuclei. Nat. Rev. Neurosci..

[b0580] Sengupta A., Bocchio M., Bannerman D.M., Sharp T., Capogna M. (2017). Control of amygdala circuits by 5-HT neurons via 5-HT and glutamate cotransmission. J. Neurosci..

[b0585] Shine J.M., Lewis L.D., Garrett D.D., Hwang K. (2023). The impact of the human thalamus on brain-wide information processing. Nat. Rev. Neurosci..

[b0590] Siddiqi S.H., Khosravani S., Rolston J.D., Fox M.D. (2024). The future of brain circuit-targeted therapeutics. Neuropsychopharmacology.

[b0595] Sieveritz B., García-Muñoz M., Arbuthnott G.W. (2019). Thalamic afferents to prefrontal cortices from ventral motor nuclei in decision-making. Eur. J. Neurosci..

[b0600] Simani L., Raminfard S., Asadollahi M., Roozbeh M., Ryan F., Rostami M. (2020). Neurochemicals of limbic system and thalamofrontal cortical network: are they different between patients with idiopathic generalized epilepsy and psychogenic nonepileptic seizure?. Epilepsy Behav..

[b0605] Singh K., García-Gomar M.G., Bianciardi M. (2021). Probabilistic atlas of the mesencephalic reticular formation, isthmic reticular formation, microcellular tegmental nucleus, ventral tegmental area nucleus complex, and caudal-rostral linear raphe nucleus complex in living humans from 7 tesla magnetic resonance imaging. Brain Connect..

[b0610] Smith S.M., Nichols T.E., Vidaurre D., Winkler A.M., Behrens T.E.J., Glasser M.F., Ugurbil K., Barch D.M., Van Essen D.C., Miller K.L. (2015). A positive-negative mode of population covariation links brain connectivity, demographics and behavior. Nat. Neurosci..

[b0615] Snow J.C., Allen H.A., Rafal R.D., Humphreys G.W. (2009). Impaired attentional selection following lesions to human pulvinar: evidence for homology between human and monkey. Proc. Natl. Acad. Sci..

[b0620] Sojka P., Diez I., Bareš M., Perez D.L. (2021). Individual differences in interoceptive accuracy and prediction error in motor functional neurological disorders: a DTI study. Hum. Brain Mapp..

[b0625] Sojka P., Serranová T., Khalsa S.S., Perez D.L., Diez I. (2025). Altered neural processing of interoception in patients with functional neurological disorder: a task-based fMRI study. J. Neuropsychiatry Clin. Neurosci..

[b0630] Spagnolo P.A., Parker J.A., Hallett M., Horovitz S.G. (2025). Functional movement disorder is associated with abnormal interoceptive brain activity: a task-based functional MRI study. Front. Psych..

[b0635] Sterling P. (2012). Allostasis: a model of predictive regulation. Physiol. Behav..

[b0640] Su J.H., Thomas F.T., Kasoff W.S., Tourdias T., Choi E.Y., Rutt B.K., Saranathan M. (2019). Thalamus optimized multi atlas segmentation (THOMAS): fast, fully automated segmentation of thalamic nuclei from structural MRI. Neuroimage.

[b0650] Theriault J.E., Katsumi Y., Reimann H.M., Zhang J., Deming P., Dickerson B.C., Quigley K.S., Barrett L.F. (2025). It's not the thought that counts: Allostasis at the core of brain function. Neuron.

[b0655] Theysohn J.M., Kraff O., Eilers K., Andrade D., Gerwig M., Timmann D., Schmitt F., Ladd M.E., Ladd S.C., Bitz A.K. (2014). Vestibular effects of a 7 Tesla MRI examination compared to 1.5 T and 0 T in healthy volunteers. PLoS One.

[b0660] Thomas E.R., Haarsma J., Nicholson J., Yon D., Kok P., Press C. (2024). Predictions and errors are distinctly represented across V1 layers. Curr. Biol..

[b0665] Timbie C., Barbas H. (2015). Pathways for emotions: specializations in the amygdalar, mediodorsal thalamic, and posterior orbitofrontal network. J. Neurosci..

[b0670] Tomic A., Agosta F., Sarasso E., Petrovic I., Basaia S., Pesic D., Kostic M., Fontana A., Kostic V.S., Filippi M. (2020). Are there two different forms of functional dystonia? A multimodal brain structural MRI study. Mol. Psychiatry.

[b0675] Tourdias T., Saranathan M., Levesque I.R., Su J., Rutt B.K. (2014). Visualization of intra-thalamic nuclei with optimized white-matter-nulled MPRAGE at 7T. Neuroimage.

[b0685] Tournier J.D., Calamante F., Connelly A. (2007). Robust determination of the fibre orientation distribution in diffusion MRI: non-negativity constrained super-resolved spherical deconvolution. Neuroimage.

[b0680] Tournier J.D., Yeh C.H., Calamante F., Cho K.H., Connelly A., Lin C.P. (2008). Resolving crossing fibres using constrained spherical deconvolution: validation using diffusion-weighted imaging phantom data. Neuroimage.

[b0690] van der Kruijs S.J., Bodde N.M., Vaessen M.J., Lazeron R.H., Vonck K., Boon P., Hofman P.A., Backes W.H., Aldenkamp A.P., Jansen J.F. (2012). Functional connectivity of dissociation in patients with psychogenic non-epileptic seizures. J. Neurol. Neurosurg. Psychiatry.

[b0695] van der Kruijs S.J., Jagannathan S.R., Bodde N.M., Besseling R.M., Lazeron R.H., Vonck K.E., Boon P.A., Cluitmans P.J., Hofman P.A., Backes W.H., Aldenkamp A.P., Jansen J.F. (2014). Resting-state networks and dissociation in psychogenic non-epileptic seizures. J. Psychiatr. Res..

[b0700] Vano L.J., McCutcheon R.A., Sedlacik J., Rutigliano G., Kaar S.J., Finelli V., Lobo M.C., Berry A., Statton B., Fazlollahi A., Everall I.P., Howes O.D. (2025). The role of low subcortical iron, white matter myelin, and oligodendrocytes in schizophrenia: a quantitative susceptibility mapping and diffusion tensor imaging study. Mol. Psychiatry.

[b0705] Voelker M.N., Kraff O., Goerke S., Laun F.B., Hanspach J., Pine K.J., Ehses P., Zaiss M., Liebert A., Straub S., Eckstein K., Robinson S., Nagel A.N., Stefanescu M.R., Wollrab A., Klix S., Felder J., Hock M., Bosch D., Weiskopf N., Speck O., Ladd M.E., Quick H.H. (2021). The traveling heads 2.0: Multicenter reproducibility of quantitative imaging methods at 7 Tesla. NeuroImage.

[b0710] Voon V., Brezing C., Gallea C., Ameli R., Roelofs K., LaFrance W.C., Hallett M. (2010). Emotional stimuli and motor conversion disorder. Brain.

[b0720] Voon V., Gallea C., Hattori N., Bruno M., Ekanayake V., Hallett M. (2010). The involuntary nature of conversion disorder. Neurology.

[b0715] Voon V., Brezing C., Gallea C., Hallett M. (2011). Aberrant supplementary motor complex and limbic activity during motor preparation in motor conversion disorder. Mov. Disord..

[b0725] Vuilleumier P., Chicherio C., Assal F., Schwartz S., Slosman D., Landis T. (2001). Functional neuroanatomical correlates of hysterical sensorimotor loss. Brain.

[b0730] Walpola I.C., Mohan A., Foster S., Kozlowska K. (2025). Altered self-processing brain networks in paediatric functional neurological disorder. Neuroimage Clin.

[b0740] Weber S., Heim S., Richiardi J., Van De Ville D., Serranová T., Jech R., Marapin R.S., Tijssen M.A.J., Aybek S. (2022). Multi-centre classification of functional neurological disorders based on resting-state functional connectivity. Neuroimage Clin.

[b0735] Weber S., Bühler J., Vanini G., Loukas S., Bruckmaier R., Aybek S. (2023). Identification of biopsychological trait markers in functional neurological disorders. Brain.

[b0750] Weber S., Jungilligens J., Aybek S., Popkirov S. (2024). Locus coeruleus co-activation patterns at rest show higher state persistence in patients with dissociative seizures: a pilot study. Epilepsia Open.

[b0745] Weber S., Bühler J., Bolton T.A.W., Aybek S. (2025). Altered brain network dynamics in motor functional neurological disorders: the role of the right temporo-parietal junction. Transl. Psychiatry.

[b0755] Wegrzyk J., Kebets V., Richiardi J., Galli S., Van de Ville D., Aybek S. (2018). Identifying motor functional neurological disorder using resting-state functional connectivity. NeuroImage: Clin..

[b0760] Weis C.N., Bennett K.P., Huggins A.A., Parisi E.A., Gorka S.M., Larson C. (2022). A 7-Tesla MRI study of the periaqueductal gray: resting state and task activation under threat. Soc. Cogn. Affect. Neurosci..

[b0765] Weiskopf N., Edwards L.J., Helms G., Mohammadi S., Kirilina E. (2021). Quantitative magnetic resonance imaging of brain anatomy and in vivo histology. Nat. Rev. Phys..

[b0770] Wen G.C., Schloesser Dana M., Arensdorf Angela M., Janine S., Changhai C., Valentino Rita W., James G., Lisbeth N., St H.-C., Spruance Victoria S., Horowitz Todd F., Yolanda V., Langevin Helene M. (2021). The emerging science of interoception: sensing, integrating, interpreting, and regulating signals within the self. Trends Neurosci..

[b0775] Westlin C., Guthrie A.J., Bleier C., Finkelstein S.A., Maggio J., Ranford J., MacLean J., Godena E., Millstein D., Paredes-Echeverri S., Freeburn J., Adams C., Stephen C.D., Diez I., Perez D.L. (2025). Delineating network integration and segregation in the pathophysiology of functional neurological disorder. Brain Commun..

[b0780] Westlin C., Guthrie A.J., Bleier C., Finkelstein S.A., Maggio J., Ranford J., MacLean J., Godena E., Millstein D., Freeburn J., Adams C., Stephen C.D., Diez I., Perez D.L., Katsumi Y. (2025).

[b0785] Wilson M., Andronesi O., Barker P.B., Bartha R., Bizzi A., Bolan P.J., Brindle K.M., Choi I.Y., Cudalbu C., Dydak U., Emir U.E., Gonzalez R.G., Gruber S., Gruetter R., Gupta R.K., Heerschap A., Henning A., Hetherington H.P., Huppi P.S., Hurd R.E., Kantarci K., Kauppinen R.A., Klomp D.W.J., Kreis R., Kruiskamp M.J., Leach M.O., Lin A.P., Luijten P.R., Marjańska M., Maudsley A.A., Meyerhoff D.J., Mountford C.E., Mullins P.G., Murdoch J.B., Nelson S.J., Noeske R., Öz G., Pan J.W., Peet A.C., Poptani H., Posse S., Ratai E.M., Salibi N., Scheenen T.W.J., Smith I.C.P., Soher B.J., Tkáč I., Vigneron D.B., Howe F.A. (2019). Methodological consensus on clinical proton MRS of the brain: Review and recommendations. Magn. Reson. Med..

[b0790] Wisse L.E.M., Chételat G., Daugherty A.M., de Flores R., la Joie R., Mueller S.G., Stark C.E.L., Wang L., Yushkevich P.A., Berron D., Raz N., Bakker A., Olsen R.K., Carr V.A. (2021). Hippocampal subfield volumetry from structural isotropic 1 mm(3) MRI scans: a note of caution. Hum. Brain Mapp..

[b0795] Ye S., Dave A., Salami A., Ziaei M. (2025). Frontoparietal functional dedifferentiation during naturalistic movie watching among older adults at risk of emotional vulnerability. Neurobiol. Aging.

[b0800] Young, Alexandra L., Razvan V. Marinescu, Neil P. Oxtoby, Martina Bocchetta, Keir Yong, Nicholas C. Firth, David M. Cash, David L. Thomas, Katrina M. Dick, Jorge Cardoso, John van Swieten, Barbara Borroni, Daniela Galimberti, Mario Masellis, Maria Carmela Tartaglia, James B. Rowe, Caroline Graff, Fabrizio Tagliavini, Giovanni B. Frisoni, Robert Laforce, Elizabeth Finger, Alexandre de Mendonça, Sandro Sorbi, Jason D. Warren, Sebastian Crutch, Nick C. Fox, Sebastien Ourselin, Jonathan M. Schott, Jonathan D. Rohrer, Daniel C. Alexander, Christin Andersson, Silvana Archetti, Andrea Arighi, Luisa Benussi, Giuliano Binetti, Sandra Black, Maura Cosseddu, Marie Fallström, Carlos Ferreira, Chiara Fenoglio, Morris Freedman, Giorgio G. Fumagalli, Stefano Gazzina, Roberta Ghidoni, Marina Grisoli, Vesna Jelic, Lize Jiskoot, Ron Keren, Gemma Lombardi, Carolina Maruta, Lieke Meeter, Simon Mead, Rick van Minkelen, Benedetta Nacmias, Linn Öijerstedt, Alessandro Padovani, Jessica Panman, Michela Pievani, Cristina Polito, Enrico Premi, Sara Prioni, Rosa Rademakers, Veronica Redaelli, Ekaterina Rogaeva, Giacomina Rossi, Martin Rossor, Elio Scarpini, David Tang-Wai, Hakan Thonberg, Pietro Tiraboschi, Ana Verdelho, Michael W. Weiner, Paul Aisen, Ronald Petersen, Clifford R. Jack, William Jagust, John Q. Trojanowki, Arthur W. Toga, Laurel Beckett, Robert C. Green, Andrew J. Saykin, John Morris, Leslie M. Shaw, Zaven Khachaturian, Greg Sorensen, Lew Kuller, Marc Raichle, Steven Paul, Peter Davies, Howard Fillit, Franz Hefti, Davie Holtzman, M. Marcel Mesulam, William Potter, Peter Snyder, Adam Schwartz, Tom Montine, Ronald G. Thomas, Michael Donohue, Sarah Walter, Devon Gessert, Tamie Sather, Gus Jiminez, Danielle Harvey, Matthew Bernstein, Paul Thompson, Norbert Schuff, Bret Borowski, Jeff Gunter, Matt Senjem, Prashanthi Vemuri, David Jones, Kejal Kantarci, Chad Ward, Robert A. Koeppe, Norm Foster, Eric M. Reiman, Kewei Chen, Chet Mathis, Susan Landau, Nigel J. Cairns, Erin Householder, Lisa Taylor-Reinwald, Virginia Lee, Magdalena Korecka, Michal Figurski, Karen Crawford, Scott Neu, Tatiana M. Foroud, Steven Potkin, Li Shen, Kelley Faber, Sungeun Kim, Kwangsik Nho, Leon Thal, Neil Buckholtz, Marylyn Albert, Richard Frank, John Hsiao, Jeffrey Kaye, Joseph Quinn, Betty Lind, Raina Carter, Sara Dolen, Lon S. Schneider, Sonia Pawluczyk, Mauricio Beccera, Liberty Teodoro, Bryan M. Spann, James Brewer, Helen Vanderswag, Adam Fleisher, Judith L. Heidebrink, Joanne L. Lord, Sara S. Mason, Colleen S. Albers, David Knopman, Kris Johnson, Rachelle S. Doody, Javier Villanueva-Meyer, Munir Chowdhury, Susan Rountree, Mimi Dang, Yaakov Stern, Lawrence S. Honig, Karen L. Bell, Beau Ances, Maria Carroll, Sue Leon, Mark A. Mintun, Stacy Schneider, Angela Oliver, Daniel Marson, Randall Griffith, David Clark, David Geldmacher, John Brockington, Erik Roberson, Hillel Grossman, Effie Mitsis, Leyla de Toledo-Morrell, Raj C. Shah, Ranjan Duara, Daniel Varon, Maria T. Greig, Peggy Roberts, Marilyn Albert, Chiadi Onyike, Daniel D’Agostino, Stephanie Kielb, James E. Galvin, Brittany Cerbone, Christina A. Michel, Henry Rusinek, Mony J. de Leon, Lidia Glodzik, Susan De Santi, P. Murali Doraiswamy, Jeffrey R. Petrella, Terence Z. Wong, Steven E. Arnold, Jason H. Karlawish, David Wolk, Charles D. Smith, Greg Jicha, Peter Hardy, Partha Sinha, Elizabeth Oates, Gary Conrad, Oscar L. Lopez, MaryAnn Oakley, Donna M. Simpson, Anton P. Porsteinsson, Bonnie S. Goldstein, Kim Martin, Kelly M. Makino, M. Saleem Ismail, Connie Brand, Ruth A. Mulnard, Gaby Thai, Catherine Mc-Adams-Ortiz, Kyle Womack, Dana Mathews, Mary Quiceno, Ramon Diaz-Arrastia, Richard King, Myron Weiner, Kristen Martin-Cook, Michael DeVous, Allan I. Levey, James J. Lah, Janet S. Cellar, Jeffrey M. Burns, Heather S. Anderson, Russell H. Swerdlow, Liana Apostolova, Kathleen Tingus, Ellen Woo, Daniel Hs Silverman, Po H. Lu, George Bartzokis, Neill R. Graff-Radford, Francine Parfitt, Tracy Kendall, Heather Johnson, Martin R. Farlow, Ann Marie Hake, Brandy R. Matthews, Scott Herring, Cynthia Hunt, Christopher H. van Dyck, Richard E. Carson, Martha G. MacAvoy, Howard Chertkow, Howard Bergman, Chris Hosein, Bojana Stefanovic, Curtis Caldwell, Ging-Yuek Robin Hsiung, Howard Feldman, Benita Mudge, Michele Assaly, Andrew Kertesz, John Rogers, Charles Bernick, Donna Munic, Diana Kerwin, Marek-Marsel Mesulam, Kristine Lipowski, Chuang-Kuo Wu, Nancy Johnson, Carl Sadowsky, Walter Martinez, Teresa Villena, Raymond Scott Turner, Kathleen Johnson, Brigid Reynolds, Reisa A. Sperling, Keith A. Johnson, Gad Marshall, Meghan Frey, Barton Lane, Allyson Rosen, Jared Tinklenberg, Marwan N. Sabbagh, Christine M. Belden, Sandra A. Jacobson, Sherye A. Sirrel, Neil Kowall, Ronald Killiany, Andrew E. Budson, Alexander Norbash, Patricia Lynn Johnson, Joanne Allard, Alan Lerner, Paula Ogrocki, Leon Hudson, Evan Fletcher, F. T. D. Initiative The Genetic, and Initiative The Alzheimer’s Disease Neuroimaging. 2018. 'Uncovering the heterogeneity and temporal complexity of neurodegenerative diseases with Subtype and Stage Inference', *Nature Communications*, 9: 4273. 10.1038/s41467-018-05892-0 PMC6189176 30323170

[b0810] Zhang H., Schneider T., Wheeler-Kingshott C.A., Alexander D.C. (2012). NODDI: Practical in vivo neurite orientation dispersion and density imaging of the human brain. Neuroimage.

[b0805] Zhang H., Zhu Z., Ma W.X., Kong L.X., Yuan P.C., Bu L.F., Han J., Huang Z.L., Wang Y.Q. (2024). The contribution of periaqueductal gray in the regulation of physiological and pathological behaviors. Front. Neurosci..

[b0815] Zhou H., Schafer R.J., Desimone R. (2016). Pulvinar-cortex interactions in vision and attention. Neuron.

