# Supplementary material for: Ultra-high field brain MRI for functional neurological disorder: opportunities and challenges
Source: Neuroimage Clin. 2026 Feb 19;50:103972. doi: 10.1016/j.nicl.2026.103972 (PMC13010964; doi:10.1016/j.nicl.2026.103972)
Supplement: Supplementary Box 1 — Technical considerations and artefact mitigation at 7T. [file mmc1.docx]

## **Supplementary Box 1:** Technical considerations and artefact mitigation at 7T MRI

UHF MRI at 7T provides substantial gains in SNR and spatial resolution, but simultaneously amplifies physical effects that can degrade image uniformity, geometric fidelity, and temporal stability if not explicitly addressed (Balchandani and Naidich 2015; Barisano et al. 2019).

**B₁⁺ inhomogeneity.**

At 7T, the RF wavelength in tissue (~12 cm) approaches head dimensions, producing spatially non-uniform transmit fields and flip-angle variation. This results in regional signal loss or contrast heterogeneity, particularly in inferior and lateral brain regions (Balchandani and Naidich 2015; Barisano et al. 2019).

**B₀ inhomogeneity.**

Susceptibility differences between tissues and at air–tissue interfaces scale with field strength, causing local frequency shifts, geometric distortion, and signal loss. These effects are most prominent in orbitofrontal cortex, medial temporal lobe, insula, and brainstem regions (Balchandani and Naidich 2015; Barisano et al. 2019).

**Chemical-shift artefacts.**

The water-fat resonance offset increases linearly with B₀ (~300 Hz at 7T), increasing spatial misregistration in gradient-echo (GRE) and echo-planar imaging (EPI) unless bandwidth and pulse design are optimized (Balchandani and Naidich 2015).

**Relaxation-time changes.**

T₁ relaxation times lengthen at UHF, altering optimal repetition time (TR) and flip-angle combinations for T₁-weighted imaging. In contrast, apparent T₂ and particularly T₂* shorten due to enhanced susceptibility effects, increasing vulnerability to signal loss in GRE- and EPI-based acquisitions (Balchandani and Naidich 2015; Barisano et al. 2019).

**Specific Absorption Rate (SAR).**

RF energy deposition increases with field strength and flip angle, making SAR a primary constraint at 7T. Sequence design must therefore balance contrast, coverage, and repetition rate against regulatory limits (Padormo et al. 2016; Balchandani and Naidich 2015).

**Susceptibility effects and blooming.**

Enhanced susceptibility contrast at 7T improves visualization of iron-rich structures but also increases local field gradients, leading to signal loss and geometric distortion in GRE-based sequences. These effects can be exploited quantitatively using susceptibility-based approaches when appropriately modelled (Barisano et al. 2019).

**Physiological and temporal instabilities.**

Physiological noise from cardiac and respiratory sources increases with signal amplitude at UHF, reducing temporal SNR and affecting functional and time-series analyses (Fagan et al. 2021).

**Mitigation strategies.**

Parallel transmission (pTx) systems enable spatial homogenization of the B₁⁺ field, while dielectric pads locally improve transmit efficiency. Advanced B₀ shimming strategies reduce geometric distortion, particularly in EPI acquisitions. Low-SAR pulse designs, spin-echo-based sequences, multi-echo acquisitions, and physiological noise correction methods improve image stability and robustness at 7T (Padormo et al. 2016; Barisano et al. 2019; Fagan et al. 2021; Özütemiz et al. 2023).

**Multi-site harmonization and reproducibility considerations.**

Beyond physics-related artefacts, multi-site studies introduce additional sources of variability, including differences in hardware configurations (vendor platform, RF coil design, availability/implementation of parallel transmission), as well as site-specific shimming and calibration workflows and sequence parameterization. These factors can increase between-site variance and complicate pooling unless harmonization is planned a priori (Clarke et al. 2020). Consistent with this, multi-center “traveling-head” studies (i.e., the same participants scanned across multiple sites) show that quantitative brain imaging at 7T can be highly reproducible across sites, while still revealing systematic differences linked to calibration and RF coil/B₁ procedures (Voelker et al. 2021). A practical strategy to support comparability over time and between sites is the use of standardized system phantoms and calibration routines, alongside shared preprocessing and quality control pipelines (Keenan et al. 2018). As demonstrated by disease-focused networks, these measures enable feasible multi-site 7T imaging while maintaining protocol consistency and quality assurance (Düzel et al. 2019).

*Abbreviations: EPI, echo-planar imaging; GRE, gradient-echo imaging; pTx, parallel transmission; RF, radio frequency; SAR, specific absorption rate; SNR, signal-to-noise ratio; TR, repetition time.*

**References:**

Balchandani, P., and T. P. Naidich. 2015. 'Ultra-High-Field MR Neuroimaging', *AJNR Am J Neuroradiol*, 36: 1204–15.

Barisano, G., F. Sepehrband, S. Ma, K. Jann, R. Cabeen, D. J. Wang, A. W. Toga, and M. Law. 2019. 'Clinical 7 T MRI: Are we there yet? A review about magnetic resonance imaging at ultra-high field', *Br J Radiol*, 92: 20180492.

Clarke, William T., Olivier Mougin, Ian D. Driver, Catarina Rua, Andrew T. Morgan, Michael Asghar, Stuart Clare, Susan Francis, Richard G. Wise, Christopher T. Rodgers, Adrian Carpenter, Keith Muir, and Richard Bowtell. 2020. 'Multi-site harmonization of 7 tesla MRI neuroimaging protocols', *NeuroImage*, 206: 116335.

Düzel, Emrah, Julio Acosta-Cabronero, David Berron, Geert Jan Biessels, Isabella Björkman-Burtscher, Michel Bottlaender, Richard Bowtell, Mark v Buchem, Arturo Cardenas-Blanco, Fawzi Boumezbeur, Dennis Chan, Stuart Clare, Mauro Costagli, Ludovic de Rochefort, Ariane Fillmer, Penny Gowland, Oskar Hansson, Jeroen Hendrikse, Oliver Kraff, Mark E. Ladd, Itamar Ronen, Esben Petersen, James B. Rowe, Hartwig Siebner, Tony Stoecker, Sina Straub, Michela Tosetti, Kamil Uludag, Alexandre Vignaud, Jaco Zwanenburg, and Oliver Speck. 2019. 'European Ultrahigh-Field Imaging Network for Neurodegenerative Diseases (EUFIND)', *Alzheimer's & Dementia: Diagnosis, Assessment & Disease Monitoring*, 11: 538–49.

Fagan, A. J., A. K. Bitz, I. M. Björkman-Burtscher, C. M. Collins, V. Kimbrell, and A. J. E. Raaijmakers. 2021. '7T MR Safety', *J Magn Reson Imaging*, 53: 333–46.

Keenan, Kathryn E., Maureen Ainslie, Alex J. Barker, Michael A. Boss, Kim M. Cecil, Cecil Charles, Thomas L. Chenevert, Larry Clarke, Jeffrey L. Evelhoch, Paul Finn, Daniel Gembris, Jeffrey L. Gunter, Derek L. G. Hill, Clifford R. Jack Jr, Edward F. Jackson, Guoying Liu, Stephen E. Russek, Samir D. Sharma, Michael Steckner, Karl F. Stupic, Joshua D. Trzasko, Chun Yuan, and Jie Zheng. 2018. 'Quantitative magnetic resonance imaging phantoms: A review and the need for a system phantom', *Magnetic Resonance in Medicine*, 79: 48–61.

Padormo, F., A. Beqiri, J. V. Hajnal, and S. J. Malik. 2016. 'Parallel transmission for ultrahigh-field imaging', *NMR Biomed*, 29: 1145–61.

Voelker, Maximilian N., Oliver Kraff, Steffen Goerke, Frederik B. Laun, Jannis Hanspach, Kerrin J. Pine, Philipp Ehses, Moritz Zaiss, Andrzej Liebert, Sina Straub, Korbinian Eckstein, Simon Robinson, Armin N. Nagel, Maria R. Stefanescu, Astrid Wollrab, Sabrina Klix, Jörg Felder, Michael Hock, Dario Bosch, Nikolaus Weiskopf, Oliver Speck, Mark E. Ladd, and Harald H. Quick. 2021. 'The traveling heads 2.0: Multicenter reproducibility of quantitative imaging methods at 7 Tesla', *NeuroImage*, 232: 117910.

Özütemiz, C., M. White, W. Elvendahl, Y. Eryaman, M. Marjańska, G. J. Metzger, R. Patriat, J. Kulesa, N. Harel, Y. Watanabe, A. Grant, G. Genovese, and Z. Cayci. 2023. 'Use of a Commercial 7-T MRI Scanner for Clinical Brain Imaging: Indications, Protocols, Challenges, and Solutions-A Single-Center Experience', *AJR Am J Roentgenol*, 221: 788–804.
